# Direct Ink Writing Based 4D Printing of Materials and Their Applications

**DOI:** 10.1002/advs.202001000

**Published:** 2020-06-29

**Authors:** Xue Wan, Lan Luo, Yanju Liu, Jinsong Leng

**Affiliations:** ^1^ Center for Composite Materials and Structures Harbin Institute of Technology Harbin 150080 P. R. China; ^2^ Department of Astronautical Science and Mechanics Harbin Institute of Technology Harbin 150001 P. R. China

**Keywords:** 4D printing, direct ink writing, hydrogels, liquid crystal elastomers, shape memory polymers

## Abstract

4D printing has attracted academic interest in the recent years because it endows static printed structures with dynamic properties with the change of time. The shapes, functionalities, or properties of the 4D printed objects could alter under various stimuli such as heat, light, electric, and magnetic field. Briefly, 4D printing is the development of 3D printing with the fourth dimension of time. Among the fabrication techniques that have been employed for 4D printing, the direct ink writing technique shows superiority due to its open source for various types of materials. Herein, the state‐of‐the‐art achievements about the topic of 4D printing through direct ink writing are summarized. The types of materials, printing strategies, actuated methods, and their potential applications are discussed in detail. To date, most efforts have been devoted to shape‐shifting materials, including shape memory polymers, hydrogels, and liquid crystal elastomers, showing great prospects in areas ranging from the biomedical field to robotics. Finally, the current challenges and outlook toward 4D printing based on direct ink writing are also pointed out to leave open a significant space for future innovation.

## Graphical abstract

4D printing has attracted wide attention due to its potential to extend the design space beyond complex geometries with the change of time. Recent achievements in 4D printing based on the direct ink writing technique are reviewed in detail, including types of materials, their functionalities, and applications. Current challenges, future developing trends, and directions are also presented.

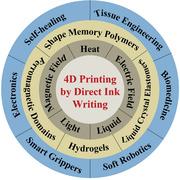

## 1 Introduction

3D printing or additive manufacturing, which was invented in 1986, is a technique that creates complex and customer‐designed objects without the use of any molds.^[^ ^1^, ^2^, ^3^ ^]^ This technique has overcome the limitations of traditional processing methods and driven great innovations in various applications ranging from soft robotics to tissue engineering, thus becoming one of the most important technologies in manufacturing industry.^[^ ^4^, ^5^ ^]^ 4D printing, a developed term raised by Tibbits at TED talk, first referred to a new technique that the shapes of printed 3D objects changed with time.^[^ ^6^, ^7^ ^]^ Recently, the definition has been extended to not just the shapes but also functionalities or properties that could alter with time under an external stimulus, such as heat,^[^ ^8^, ^9^ ^]^ electric field,^[^ ^10^ ^]^ magnetic field,^[^ ^11^ ^]^ light,^[^ ^12^ ^]^ and liquid,^[^ ^13^ ^]^ etc. The fabrication methods of 4D printing are the same as that of 3D printing, including stereolithography (SLA),^[^ ^14^ ^]^ digital light processing (DLP),^[^ ^15^ ^]^ fused deposition modeling (FDM),^[^ ^12^ ^]^ direct ink writing (DIW),^[^ ^16^ ^]^ and PolyJet.^[^ ^17^ ^]^

**Figure 1:**
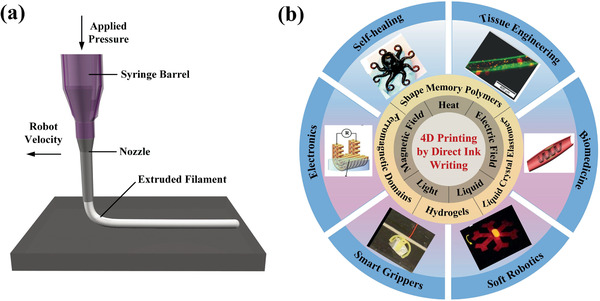
Brief summary of 4D printing by direct ink writing. a) Schematic illustration of direct ink writing. Reproduced with permission.^[^ ^150^ ^]^ Copyright 2006, Wiley‐VCH. b) The important elements evolved in 4D printing by direct ink writing. The actuation methods: heat, electric field, magnetic field, light, and liquid. Major categories of materials: shape memory polymers, liquid crystal elastomers, hydrogels, and ferromagnetic domains. Representative functionality (reproduced with permission.^[^ ^137^ ^]^ Copyright 2017, Royal Society of Chemistry) and applications: tissue engineering (reproduced with permission.^[^ ^94^ ^]^ Copyright 2019, Wiley‐VCH), biomedicine (reproduced with permission.^[^ ^16^ ^]^ Copyright 2017, American Chemical Society), electronics (reproduced with permission.^[^ ^60^ ^]^ Copyright 2019, Elsevier), smart grippers (reproduced with permission.^[^ ^84^ ^]^ Copyright 2019, Wiley‐VCH), and soft robotics (reproduced with permission.^[^ ^11^ ^]^ Copyright 2018, Nature Publishing Group).

During these 4D printing methods, SLA and DLP could realize both commercial and lab‐made materials with fast printing speed and excellent printing resolution.^[^ ^18^, ^19^ ^]^ However, they are only applicable to photocurable resins and hard to construct composite materials with not‐transparent fillers because of the restriction of optical requirement for photocuring. Accordingly, the method to actuate 4D printed structures printed by SLA and DLP is usually confined to heat stimulus. FDM is a mature printing technique that has been widely used in both industry and laboratory with relatively fast printing speed.^[^ ^20^, ^21^ ^]^ The actuation methods are various (such as heat, electric field, magnetic field, and light) according to the addition of functional fillers into printer filaments.^[^ ^22^ ^]^ However, the printing resolution is relatively low and the preparation of printing filaments usually costs too much waste of materials due to the characteristics of extruding machines. Besides, the FDM printer works by melting printer filaments above the melting temperature, which may cause invalidation and degradation of temperature‐sensitive components in the printer filaments, thus limiting their ultimate applications where require long period of stability. In recent years, Stratasys has produced some popular commercial printers based on PolyJet technique and utilized them to print multimaterial. This technique realizes multimaterial printing such as composite shape memory polymers (SMPs) and composite hydrogels with widely tunable properties. However, the cost of printer is quite expensive and the ink is usually provided by Stratasys with unknown compositions but primarily acrylic‐based photopolymers, thus limiting their choices of materials. Similar to SLA and DLP, the functional not‐transparent fillers impede the light absorption of photocurable resin during printing. Until now, the stimulus to actuate 4D structures printed by PolyJet is usually heat.^[^ ^23^, ^24^ ^]^ The DIW technique refers to a printing method based on extrusion through a nozzle under pressure, utilizing a computer‐controlled robot to move the dispenser filled with printed ink to construct geometries layer‐by‐layer, as shown in **Figure** **1**a.^[^ ^25^, ^26^ ^]^ The use of micronozzles in DIW enables to achieve high printing resolution, which is favorable when considering radio frequency and autonomously powered microdevices.^[^ ^27^, ^28^ ^]^ Compared to other printing methods, the DIW technique shows superiority due to free choices of materials, small amount of raw materials, open source and feasibility for multimaterial printing.^[^ ^29^ ^]^ It is a laboratory‐friendly technique especially when printing nanocomposites with different content of nanofillers or nanoparticles, regardless of whether they are transparent or not. The expense of printing process is cheap and the choice of barrel volume and nozzle size is quite flexible according to the demands of customers. It is easy to build such a printing platform by labs themselves as the working principle is easy, consisting of a three‐axis platform, computer, and dispenser.^[^ ^1^, ^30^ ^]^ So far, DIW has successfully printed various types of materials, such as metal particles,^[^ ^31^ ^]^ polymers,^[^ ^32^ ^]^ ceramics,^[^ ^33^ ^]^ and multimaterial.^[^ ^34^ ^]^ In the DIW technique, the viscosity of ink needs to be carefully regulated because it is required to possess specific rheological performance.^[^ ^35^, ^36^, ^37^ ^]^ The vital rheological parameters for a given ink design include its apparent viscosity, yield stress under shear and compression, and viscoelastic properties (i.e., the shear loss and elastic moduli), which are tailored for the DIW technique of interest.^[^ ^25^ ^]^ The prerequisite for DIW‐based 4D printing is to design a viscoelastic ink possessing both shear‐thinning behavior and a rapid pseudoplastic to dilatant recovery.^[^ ^38^, ^39^ ^]^ A shear‐thinning behavior refers to a phenomenon that viscosity decreases with an increase of shear force inside the nozzle, which is important for the DIW fabrication of 3D structures.^[^ ^29^ ^]^ The shear‐thinning performance facilitates extrusion of inks from fine or micronozzles under pressure. On the other hand, once the ink is out of nozzle, high shear elastic modulus and shear yield strength would recover because the shear force sharply decreases. This results in shape retention after deposition. Both of the two sides help remain filamentary shape, which is necessary to construct complicated structures layer‐by‐layer. Although viscosity adjustment is required for DIW, there have been many methods to regulate it to meet the demands for successful printing, such as adding some additives to control rheology and controlling printing temperature. According to these advantages of DIW, it has been applied for 4D printing using various materials, especially shape‐shifting materials, showing great prospects in fields ranging from biomedicine to robotics.

Briefly, 4D printing is the combination of 3D printed structures with time‐evolving physical properties (shape, functionality, dimension, etc.), thus the types of printed materials are of significant importance. Figure 1b summarizes the recent progress of 4D printing by the DIW technique using different types of materials and their potential applications in biomedical field, electronics, soft robotics, and smart actuators. The most commonly used materials in 4D printing are shape‐shifting materials, which are mainly categorized into SMPs and shape‐changing materials. Liquid crystal elastomers (LCEs) and hydrogels are two major types of shape‐changing materials. Unlike SMPs, their shapes could change with time when a stimulus is applied and once the stimulus is terminated, they would return to their original shapes. SMPs involve two steps: one is a programming step to determine temporary shapes and the other is a recovering process under an external stimulus. In addition to these materials with intrinsic shape‐changing properties, the preset of localized stress or strain during the printing process is another approach to exhibit shape change over time.^[^ ^24^, ^40^, ^41^ ^]^ The flexible DIW technique enables free choice of materials loaded with active components to be applied in various areas ranging from soft robotics to biomedicine. Recently, there are some reviews of 4D printing standing at different views.^[^ ^40^, ^41^, ^42^, ^43^, ^44^, ^45^, ^46^, ^47^ ^]^ For example, there is a point of view from various types of shape‐shifting behaviors, mechanisms, and mathematics of 4D printed structures.^[^ ^40^ ^]^ Some emphasizes on materials system of 4D printing, such as single and multiple materials with different mechanisms.^[^ ^41^ ^]^ Some concentrates on structural design and shape memory polymers applied in tissue and organ regeneration.^[^ ^42^ ^]^ The shape memory effects of shape memory materials behind 4D printing along with actuation stimuli are mainly interpreted by Rastogi and Kandasubramanian.^[^ ^43^ ^]^ Others reviewed the 4D printing from perspectives of approaches and applications,^[^ ^44^ ^]^ printing technologies,^[^ ^45^ ^]^ reversible transformation,^[^ ^46^ ^]^ and smart biomaterials for 4D bioprinting.^[^ ^47^ ^]^ In this work, we review the state‐of‐the‐art advancements of 4D printing from a new perspective of DIW printing. We summarize various types of materials suitable for the DIW technique, their functionalities and point out the potential applications. To take more advantages of this research, the current challenges and future outlook of DIW‐based 4D printing are discussed. It is expected that the insights provided toward this topic could promote the further developments of 4D printing and spur innovation.

## 2 4D Printing of Shape‐Shifting Materials

### 2.1 SMPs

SMPs are a type of stimuli‐responsive smart materials, showing shape memory behavior from temporary shapes to their original shapes when triggered by an external stimulus.^[^ ^48^, ^49^, ^50^ ^]^ The dynamic shape recovery process of 3D printed SMPs shows time‐dependent shape‐changing behavior, also called as 4D printing.^[^ ^51^, ^52^ ^]^ The thermal‐responsive SMPs are the most widely investigated among all SMPs because of their high tunability of transition temperature, optical, mechanical properties and convenience of triggering shape memory.^[^ ^53^, ^54^, ^55^ ^]^

#### 2.1.1 Thermoplastic and Partially Crosslinking SMPs

**Figure 2:**
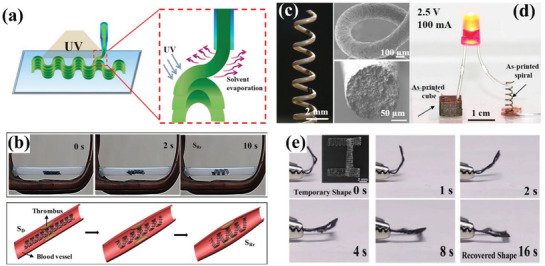
4D printing of thermoplastic and partially crosslinking SMPs via DIW. a) Schematic diagram of constructing 3D structures using PLA‐based inks layer‐by‐layer under UV irradiation. b) Self‐expanding behavior of printed PLA/Fe_3_O_4_ scaffold in response to a 30 kHz alternating magnetic field and the schematic diagram to support a narrow vessel. a,b) Reproduced with permission.^[^ ^16^ ^]^ Copyright 2017, American Chemical Society. c) Optical and SEM images of a freeform 3D printed spiral using PLA/Ag@CNFs ink. d) High electrical conductivity of PLA/Ag@CNFs nanocomposites as the printed components lighted up an LED light under a 2.5 V voltage. c,d) Reproduced with permission.^[^ ^56^ ^]^ Copyright 2017, American Chemical Society. e) Electro‐responsive shape transformation of a U‐shaped scaffold printed by PLMC/CNT ink under 25 V within 16 s. Reproduced with permission.^[^ ^60^ ^]^ Copyright 2019, Elsevier.

UV crosslinking polylactic acid (PLA)‐based inks were directly printed into shape‐changing structures with thermally and remotely actuated behavior.^[^ ^16^ ^]^ **Figure** **2**a illustrates the schematic diagram to construct 3D structures, which is realized by fast evaporation of dichloromethane (DCM). By introducing a UV photointiator, PLA formed crosslinking networks during printing under UV irradiation, thus the shape memory property was enhanced. The various structures such as spiral, wavy‐ and flower‐like multilayers showed the shape transition of 3D–1D–3D, 3D–2D–3D, and 3D–3D–3D. The addition of iron oxide into crosslinking PLA endowed the 3D printed structures with magnetically and remotely response. Then the scaffold was folded into a temporary spiral shape and placed in a tube, which restricted full shape recovery. Triggered by a 30 kHz alternating magnetic field, the scaffold showed self‐expanding behavior within 10 s as illustrated in Figure 2b. The restrictive shape‐changing behavior exhibited great potential as self‐expandable stents in biomedical field. Wei et al. further printed highly conductive multimaterial composites consisting of hybrid silver‐coated carbon nanofibers (Ag@CNFs)/PLA.^[^ ^56^ ^]^ The Ag@CNFs combined the high aspect ratio of CNF with low contact resistance of Ag.^[^ ^57^ ^]^ The printed nanocomposites possessed a low percolation threshold of <6 vol% and high volume conductivity of >2 × 10^5^ S m^−1^. The fast evaporation of DCM facilitated to print freeform 3D spiral at room temperature, as depicted in Figure 2c. When connected the printed spiral and cubic scaffold into a circuit, the high electrical conductivity enabled the light‐emitting diode (LED) light to illuminate under 2.5 V, as shown in Figure 2d.

Since an important drawback of shape memory PLA is the high glass transition temperature (*T*_g_ ≈ 61 °C) which restricts its biomedical application, Wan et al. proposed a similar DIW method to print shape memory poly(d,l‐lactide‐*co*‐trimethylene carbonate) (PLMC).^[^ ^58^ ^]^ PLMC is a copolymer with the molar ratio of d,l‐lactide:trimethylene carbonate at 80:20. The introduction of soft segment of trimethylene carbonate reduces *T*_g_ of PLMC at 49 °C. They found that the 4D printed PLMC exhibited shape‐changing behavior at 40 and 60 °C within 35 and 0.5 s, respectively. The 4D printed scaffold with a maximum recovery force of 0.2 N could bear a compressive fracture force of 12.5 N, which showed potential as an intravascular stent. Considering previous reported PLMC showed high attachment and proliferation with human cells,^[^ ^59^ ^]^ they believed that this fast responsive shape transformation exhibited great potential in biomedical devices. To make 4D printed PLMC‐based structures to be actuated in electric field, Wan et al. further introduced carbon nanotubes (CNTs) into PLMC to endow it with electro‐responsive shape transformation.^[^ ^60^ ^]^ The 4D printed structure showed excellent shape‐changing behavior under 25 V within 16 s, as illustrated in Figure 2e. Then they modified PLMC into crosslinking networks (c‐PLMC) by reaction of hydrogen abstraction under UV irradiation. After UV crosslinking, the mechanical strength and solvent resistance of c‐PLMC/CNT improved significantly while the actuation temperature and volume electrical conductivity remained almost the same.

Wang et al. synthesized waterborne shape memory polyurethane (SMPU) mixed with polyethylene oxide (PEO) or gelatin in water to enhance the viscosity of the ink.^[^ ^61^ ^]^ The PEO exhibited better shape memory properties, while gelatin exhibited better biocompatibility. They found that the addition of superparamagnetic iron oxide nanoparticles (SPIO NPs) promoted osteogenic induction and shape fixity. The printed scaffolds showed faster and better shape recovery properties in water than in an oven. Zolfagharian et al. 4D printed photoresponsive structures consisting of shape memory polystyrene, chitosan, and carbon black.^[^ ^62^ ^]^ They first prestretched polystyrene above *T*_g_ ≈ 102 °C and fixed the elongated temporary shape below *T*_g_. The addition of chitosan and carbon black served as adhesive and transparency regulator for sequential folding of the printed hinge. This method exhibited feasibility in converting 2D simple pattern of inexpensive materials into 3D complex structures under near‐infrared (NIR) irradiation.

#### 2.1.2 Thermoset SMPs

**Figure 3:**
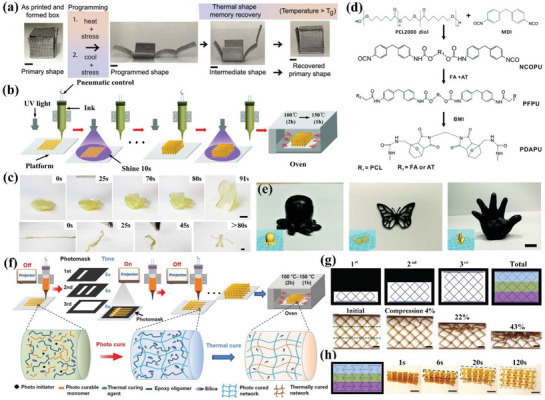
4D printing of thermoset SMPs via DIW. a) Shape programming and self‐folding transformation of a box‐like scaffold printed by ESBO/BFDGE/CNFs ink under heat stimulus (scale bars: 1 cm). Reproduced with permission.^[^ ^10^ ^]^ Copyright 2016, Nature Publishing Group. b) Schematic diagram of printing tough and isotropic epoxy assisted by UV light and post‐thermal curing. Reproduced with permission.^[^ ^64^ ^]^ Copyright 2018, Royal Society of Chemistry. c) Thermal‐responsive shape change of printed vase and toy based on semi‐IPN elastomer (scale bars: 6 mm). Reproduced with permission.^[^ ^65^ ^]^ Copyright 2018, American Chemical Society. d) Synthesis procedure of PDAPU. e) Representative complex structures printed by PDAPU (scale bar: 1 cm). d,e) Reproduced with permission.^[^ ^66^ ^]^ Copyright 2019, Royal Society of Chemistry. f) Schematic printing process of graded multimaterial assisted by dynamic photomask. g) Design of a periodic structure with three graded regions and its compressive deformation (scale bars: 10 mm). h) Sequential shape recovery process of an auxetic lattice structure within 120 s at 26 °C (scale bars: 10 mm). f–h) Reproduced with permission.^[^ ^68^ ^]^ Copyright 2019, Wiley‐VCH.

Rodriguez et al. printed 4D structures of thermoset shape memory composites (SMPCs) with complex, multimaterial architectures.^[^ ^10^ ^]^ They chose environmentally friendly and renewable epoxidized soybean oil (EBSO) as the matrix. The bisphenol F diglycidyl ether (BFDGE) was added into the matrix to increase the mechanical strength and optimize the mass ratio. The addition of CNFs tailored the rheological properties of the ink to obtain shear‐thinning performance and endowed the printed structures with electrical response. With the help of origami, the complex box‐like shape was obtained by folding the grid pattern and cured completely. Then, it was unfolded into a temporary shape. Upon reheating above *T*_g_, the box recovered to its folded shape, as shown in **Figure** **3**a. Wu et al. introduced two gas microballoon pore formers with different *T*_g_s into polydimethylsiloxane (PDMS) with silica particles (SiO_2_) to print ordered and porous 3D structures.^[^ ^63^ ^]^ They investigated the influence of shell stiffness and *T*_g_ on compressive properties of the printed porous structures. They found that under different compressive strain and reheating time, the recovery ratio of printed structures varied significantly. By combining customer‐designed geometries via DIW with the introduction of suitable microballoon, the structural shape‐changing response could be optimized. Chen et al. realized 4D printing of shape memory epoxy by UV‐assisted DIW approach.^[^ ^64^ ^]^ The printed base resin consisted of commercial photocurable acrylates with thermally curable epoxy at a weight ratio of 4/6. The SiO_2_ nanoparticles were added to modulate the viscosity to possess shear‐thinning performance. Figure 3b shows the fabrication process of tough epoxy based on UV‐assisted printing and post‐thermal curing, which enhanced the isotropic mechanical properties. The acrylates formed crosslinking networks in the UV‐assisted printing stage, followed by polymerization of epoxy in the post‐thermal curing stage. These two crosslinking networks locked together without covalent bonds, or called interpenetrated polymer network (IPN). A series of complicated structures, such as lattice, gear wheel, swirl bow and locked structures were successfully printed. The deformed structure completed shape change within 10 s at 104 °C (higher than *T*_g_ ≈ 76 °C). Similarly, Kuang et al. also developed 4D printing of highly stretchable semi‐IPN elastomer.^[^ ^65^ ^]^ It consisted of a photocurable resin, semicrystalline polycaprolactone (PCL) and fumed SiO_2_. After printing each layer, UV irradiation was utilized to trigger photopolymerization to obtain crosslinking networks. The semicrystalline PCL served as the switching segment for shape memory behavior with *T*_g_ at ≈55 °C. The shape‐changing process of 3D printed hollow vase and toy could be triggered by heat gun or hot water above this temperature, as depicted in Figure 3c.

Zhang et al. further extended the 3D and 4D printing of thermoreversible SMPU based on Diels–Alder (DA) reaction.^[^ ^66^ ^]^ They synthesized aniline trimer with strong photothermal effect as described earlier.^[^ ^67^ ^]^ The reaction of 4,4′‐diphenylmethane diisocyanate (MDI) with PCL diol was to obtain bis‐isocyanate‐terminated polyurethane (PU). Then, it reacted with a mixture of aniline trimmer and furylalcohol with an optimized molar ratio to obtain a photothermal furan‐terminated PU. Finally, bismaleimide (BMI) was added to react with furan groups to obtain photothermal DA‐reactive PUs (PDAPU) and the whole synthesis procedure is shown in Figure 3d. When heated above retro‐DA de‐crosslinking temperature, PDAPU would depolymerize to oligomers, which would facilitate extrusion from fine nozzles. The reversibility of viscosity during heating and cooling cycles made PDAPU recyclable to print again, showing potential as cost efficiently and environmentally friendly materials. Figure 3e shows some representative complex structures printed by PDAPU using a modified DIW printer. By photothermo‐mechanical programming, a temporary spiral shape could recover to its original shape under infrared (IR) illumination. While IR triggered overall shape‐changing behavior, NIR laser actuated local shape recovery in targeted exposure area. Chen et al. 4D printed graded multimaterial consisting of photocurable resin and shape memory epoxy through photomask‐assisted DIW in a two‐stage curing method as schematically shown in Figure 3f.^[^ ^68^ ^]^ The crosslinked networks of two types of polymers interlocked physically to form IPN and the mixture was modulated to perform shear‐thinning behavior by adding SiO_2_. After the ink was extruded into preset patterns each layer, it was photocured by a nearby projector using different photomasks to control the desired irradiation location with corresponding irradiation time. Thus the difference of curing degree existed within a same layer followed by the repetitive process of next layers until the graded structure was finally printed. Using this method, two representative graded structures consisting of three levels and their compressive experiments are shown in Figure 3g. It could be seen that the compressive deformation sequentially happened in the three levels because of these regions had different modulus to resist deformation. They also proved that the programming of a graded auxetic lattice structure took three steps of sequential shape recovery within 120 s above *T*_g_ ≈ 26 °C, as illustrated in Figure 3h. This research demonstrated DIW assisted by photomask endowed the multimaterial with gradient and tunable mechanical response to absorb energy and control different deformations.

### 2.2 LCEs

LCEs are another type of stimuli‐responsive smart materials with reversible and anisotropic shape‐changing performance under stimuli such as heat, light, electric, and magnetic field.^[^ ^69^, ^70^, ^71^, ^72^, ^73^, ^74^ ^]^ The two‐way actuation depends on the transformation between nematic and isotropic state at the nematic–isotropic temperature (*T*_NI_). LCEs exhibit macroscopic contraction or elongation when the temperature is above or below *T*_NI_, respectively. In 1975, de Gennes and Seances^[^ ^75^ ^]^ proposed that phase transition in LCEs led to induced stress and mechanical work, thus many researchers concentrated on making LCEs interesting candidates for actuators and artificial muscles.^[^ ^76^, ^77^ ^]^ By regulating mesophase structures within LCEs, they could obtain high strain response.^[^ ^78^ ^]^ Despite decades of research on LCEs, their potential applications have not been magnified until the advent of 4D printing in recent years.^[^ ^74^ ^]^ Besides, the new developed methods of LCE synthesis paved the way for 3D and 4D printing. 4D printing endowed LCEs with alignment of mesogens during printing process while simultaneously building complex 3D structures.

**Figure 4:**
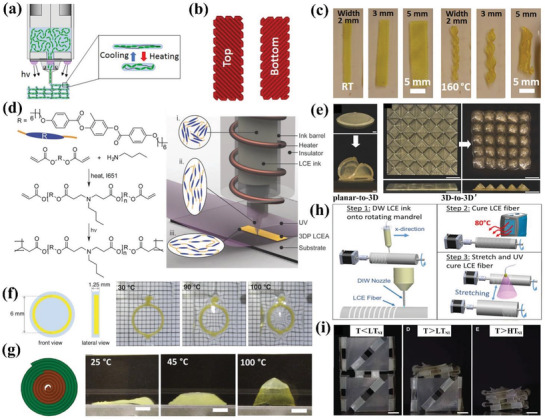
4D printing of LCEs via DIW. a) Alignment of LCE chains in the printing direction during DIW process. b) Design of printing paths in different layers to control shape transformation of c) twisted helical ribbon structures. a–c) Reproduced with permission.^[^ ^80^ ^]^ Copyright 2017, American Chemical Society. d) Synthesize procedure of LCE with modified molar ratio and schematic change of LCE molecules during printing. e) Representative planar‐to‐3D (scale bars: 1 mm) and 3D‐to‐3D′ shape transformations (scale bars: 5 mm). d,e) Reproduced with permission.^[^ ^81^ ^]^ Copyright 2018, Wiley‐VCH. f) Design of asymmetric structure of LCE/PDMS and the use of it to alter focusing properties due to contraction of LCE in elevated temperatures. Reproduced with permission.^[^ ^82^ ^]^ Copyright 2018, Wiley‐VCH. g) The utilization of printing two LCE inks with different *T*_NI_s at different locations to realize sequential shape change from disk to cone (scale bars: 5 mm). Reproduced with permission.^[^ ^84^ ^]^ Copyright 2019, Wiley‐VCH. h) Fabrication procedure of a long LCE fiber in three steps. Reproduced with permission.^[^ ^85^ ^]^ Copyright 2019, American Chemical Society. i) Sequential shape change of a manually folded 3D printed triangulated structure upon staged heating (scale bars: 1 cm). Reproduced with permission.^[^ ^86^ ^]^ Copyright 2019, American Association for the Advancement of Science.

Yuan et al. proposed the possibility of using LCE in 4D printing to achieve two‐way shape change.^[^ ^79^ ^]^ They designed a laminated structure consisting of soft substrate (printed by PolyJet), silver ink (printed by DIW), and LCE strip. The temperature to actuate LCE was controlled by conductive Joule heating of silver. When the current was on, only LCE was sensitive to elevated temperature and contracted, which caused overall bending behavior of the laminated structure. By designing the hinges consisting of laminated patterns, they achieved active two‐way shape change of airplane and Miura‐ori structure. Although the LCE was not printed directly, they proposed a method to achieve 4D printing. Ambulo et al. directly printed LCE by controlling the alignment of molecules in accordance with the direction of printing pathway, as demonstrated in **Figure** **4**a.^[^ ^80^ ^]^ The high viscous LCE ink with *T*_NI_ at 105 °C consisted of the monomer, RM 82, and the chain extender, *n*‐butylamine with the molar ratio of 1.1:1, which guaranteed the synthesized LCE chains ended with acrylate functional group. The LCE was in nematic status at a printing temperature below *T*_NI_, and the alignment of mesogens resulted from shear forces was then locked by UV photopolymerization of the acrylate end‐groups. Figure 4b,c shows by controlling various printing paths, they obtained twisted helical ribbon structures. Then they printed cylinders with zero, negative and positive Gaussian curvatures to show the shape change of azimuthal contraction and axial expansion.

Kotikian et al. utilized the same monomer and amine chain extender to synthesize LCE ink via aza‐Michael reaction except at a modified molar ratio of RM 82:*n*‐butylamine at 1:1, which decreased the *T*_NI_ to 95 °C.^[^ ^81^ ^]^ Figure 4d illustrates upon extrusion, LCE mesogens align along the printing direction and form crosslinks under UV light. With spatial programming of the printing paths, they endowed the LCE structures with planar‐to‐3D and 3D‐to‐3D′ shape transformations when heated above *T*_NI_ as shown in Figure 4e. More interestingly, the saddle shape did not recover to its original flat shape upon cooling, but inverted to an inverted saddle shape because of the residual stress produced by UV irradiation. By replacing another UV photoinitiator and modifying the molar ratio of RM 82:*n*‐butylamine of 1.01:1, Lopez‐Valdeolivas et al. printed LCE with an actuation temperature at 110 °C.^[^ ^82^ ^]^ By designing complex in‐plane patterns, they demonstrated the new shape transformations of slit, re‐entrant honeycomb and chiral structures in repeatable units based on the contraction of LCE upon heating. They further integrated the printed LCE with PDMS to fabricate an asymmetric structure to illustrate the possibility to be used as a focus lens. Under thermal stimulus, the contraction of LCE induced stress within the structure, which resulted in convex on the one side and concave on the other side. The shape change altered the focusing properties thus changing the display of light wavefront pattern, as shown in Figure 4f, which had potential as a variable optical component.

Since the actuation temperature, i.e., *T*_NI_ of LCE is high, it restricts the practical applications of 4D printed LCE. To solve this problem, Roach et al. fabricated a novel LCE ink with low *T*_NI_, which could be directly DIW printed at room temperature.^[^ ^83^ ^]^ They reduced the *T*_NI_ to 42 °C by adding a flexible dithiol spacer or 2,2′‐(ethylenedioxy) diethanethiol (EDDT) in the monomer, RM257. The alignment of LCE chains included two stages as same as the method mentioned above. They 4D printed a LCE strip and showed the reversible uniaxial actuation with a blocking stress of 385 kPa which created the possibility for soft robotics. Similar to Yuan et al.,^[^ ^79^ ^]^ they constructed smart hinges with large bending angles assisted by multimaterial and multimethod printing. To further reduce the actuation temperature and extend the practical application toward soft actuators, Saed et al. utilized a two‐step thiol‐ene reaction to obtain LCEs with *T*_NI_ ranging from ≈28 to ≈105 °C.^[^ ^84^ ^]^ In the first step, i.e., thiol‐acrylate Michael addition, they synthesized thiol‐terminated oligomers from a diacrylate reacted with three dithiols respectively to obtain different transition temperatures. The printing paths endowed the ink with orientational order and the alignment was simultaneously locked by UV crosslinking of thiol‐terminated oligomers and a trifunctional vinyl crosslinker. They investigated the influence of crosslinking strategy, crosslinking density, molecular weight of thiol spacer, and molecular weight of the mesogen on the thermomechanical properties. After optimizing these parameters, they obtained LCE with the lowest reported actuation temperature with much lower hysteresis. The 1,3‐propanedithiol‐based LCE actuator they printed was capable of performing a blocking stress higher than 500 kPa, in the same range as other main‐chain LCE systems. Finally, they printed multiple LCE inks with different *T*_NI_s at different locations within a structure to obtain sequential shape change from the disk to cone upon heating, as shown in Figure 4g. Roach et al. printed long LCE fibers based on thiol‐acrylate Michael addition and UV photo‐crosslinking by three steps depicted in Figure 4h.^[^ ^85^ ^]^ The selection of materials system was similar to their previous work.^[^ ^78^ ^]^ First, the LCE ink was deposited onto a rotating mandrel at room temperature and then cured by nearby convective heating. To regulate the viscosity of the ink, 2 wt% nanoclay was added to exhibit shear‐thinning behavior. Finally, another mandrel stretched the printed fiber under UV irradiation to fix the alignment of crosslinking networks. A LCE fiber (*T*_NI_: 65 °C) with a length of 50 mm was successfully printed and exhibited a reversible strain and blocking stress of 51% and 0.038 MPa upon heating, respectively. When twisted 8 LCE fibers together, an actuation force of ≈0.049 N was observed, showing an increase in strength. A series of intelligent textiles were obtained by knitting, sewing and weaving the LCE fibers together. By incorporating the fibers into a shirt, the wearer's increasing body temperature would cause the contraction of LCE, thus producing more pores to facilitate cooling and sweat evaporation. When the body temperature decreased, the LCE fibers would elongate thus closing the pores. Kotikian et al. printed untethered soft robotic matter, which consisted of LCE bilayers with orthogonal director alignment and soft materials.^[^ ^86^ ^]^ Two LCE inks with different *T*_NI_s were programmed at the specific area to realize sequential shape morphing process of a 3D triangulated structure under staged heating, as shown in Figure 4i. They found during the self‐propulsion actuation, the LCE bilayer generated an energy density of 29 J kg^−1^, which lifted loads more than 450 times higher than itself. Finally, they designed a flat structure which transformed to a pentagonal prism and exhibited self‐propelling behavior on a hot surface.

The above mentioned printing temperatures for LCEs were all below *T*_NI_ to make the mesogen alignment along printing direction. Recently, Zhang et al. put forward a new idea to construct 4D printed LCE structures using a single composition under a printing temperature above *T*_NI_.^[^ ^87^ ^]^ The single LCE with *T*_NI_ = 66 °C was synthesized by esterification reaction from three monomers, i.e., 4,4′‐bis(6‐hydroxyhexyloxy)‐biphenyl, 4‐(6‐hydroxyhexyloxy) cinnamic acid and *p*‐coumaric acid. After investigating the viscoelastic and rheological properties of the ink, they finally chose a printing temperature at 200 °C (within isotropic state) to ensure the viscous ink could be extruded from the micronozzle continually. The platform temperature was set at 10 °C, thus a huge temperature gradient formed between the top and bottom part in the extruded filament along the direction of thickness. In turn, it caused the difference of order parameter of mesogens, triggering reversible bending deformation upon heating or cooling. Then they found that if the printing platform was replaced by a liquid medium, the temperature difference could be eliminated. Thus there was a uniform orientation within the printed filament, which exhibited commonly contraction behavior upon heating as other literatures reported. By designing the printing areas with liquid‐assisted printing or not, Zhang et al. integrated both bending and contraction behavior within a simple 2D structure to present complex 3D cylinder shape transformation under heat stimulus.

### 2.3 Hydrogels

Shape memory hydrogels (SMHs), a kind of stimuli‐responsive soft hydrophilic materials with good biocompatibility, have developed fast in the few decades. SMH could recover from temporary shape to original or permanent shape by external stimuli such as heat, light, ultrasound, electric field, solvents, and magnetic field.^[^ ^88^, ^89^, ^90^ ^]^ In addition to SMHs, other types of hydrogels are also candidate materials for the technology of 4D printing.

#### 2.3.1 Water‐Responsive Hydrogels

**Figure 5:**
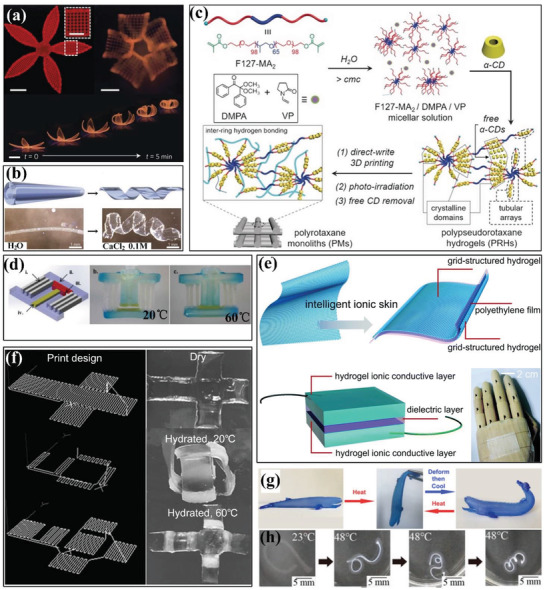
4D printing of water‐ and thermal‐responsive hydrogels via DIW. a) 4D printed flower mimicked biomimetic shape transformation based on anisotropic alignment of fibrils within the structure (scale bars: 5 mm, inset: 2.5 mm). Reproduced with permission.^[^ ^92^ ^]^ Copyright 2016, Nature Publishing Group. b) Folding and unfolding behavior of printed tube under the absence and presence of Ca^2+^ ions. Reproduced with permission.^[^ ^94^ ^]^ Copyright 2017, Wiley‐VCH. c) Fabrication procedure of polyrotaxane‐based monoliths with inter‐ring hydrogen‐bonding. Reproduced with permission.^[^ ^95^ ^]^ Copyright 2017, Wiley‐VCH. d) Thermal‐responsive poly(*N*‐isopropylacrylamide)‐based hydrogel valve to control open and closure at different temperatures. Reproduced with permission.^[^ ^99^ ^]^ Copyright 2015, Cambridge University Press. e) Structural design of a skin‐like sensor. Reproduced with permission.^[^ ^100^ ^]^ Copyright 2017, Royal Society of Chemistry. f) Folding and defolding behavior of a printed hydrogel cubic box upon cooling and heating in hydrated state. Reproduced with permission.^[^ ^101^ ^]^ Copyright 2017, Wiley‐VCH. g) Shape transformation of a printed whale‐like hydrogel composite upon thermal stimulus. Reproduced with permission.^[^ ^102^ ^]^ Copyright 2018, American Chemical Society. h) Shape‐morphing process of multilayer hydrogel from a C‐shaped structure to a 3D spring at 48 °C. Reproduced with permission.^[^ ^103^ ^]^ Copyright 2019, MDPI.

Water‐responsive hydrogels exhibit shape change such as folding and curling when immersed in water, which resulted from large absorption of water and significant swelling.^[^ ^91^ ^]^ Due to the network structure constructed by stable binding methods such as covalent bonding and coordination, the internal hydrophilic group absorbs a large amount of water, making it swell but insoluble in water. The earliest 4D printing of hydrogel was realized by Gladman et al. in 2016.^[^ ^92^ ^]^ Inspired by plant architectures, they designed the printing paths of hydrogel composite ink in 2D pattern to obtain prescribed targeted shapes when immersed in water to mimic plant behavior. The hydrogel composite consisted of *N*‐isopropylacrylamide, nanoclay, cellulose fibrils, glucose, glucose oxidase, and photoinitiator. The mechanism of shape change resulted from programmed, localized and anisotropic swelling, by controlling the alignment of cellulose fibrils during printing. The anisotropic alignment of fibrils in different directions endowed the 3D printed flower with different swelling ratios in water, thus triggering biomimetic shape transformation as illustrated in **Figure** **5**a. Subsequently, they simulated the influence of printing parameters on the final morphing structures and found it matched well with the experimental results, which was helpful for prediction of more complicated shapes based on the prescribed design.^[^ ^93^ ^]^ Kirillova et al. reported an advanced 4D biomanufacturing method combined with postprinting which realized hollow self‐folding structures with unprecedented high resolution as low as 20 µm.^[^ ^94^ ^]^ They modified two kinds of biomaterials, i.e., methacrylated alginate and hyaluronic acid with methacrylate groups and then mixed them with mouse bone marrow stromal cells to prepare the composite ink. The reversible shape change benefited from the carboxylic groups in the polymer chains, which triggered swelling or deswelling between polymer and Ca^2+^ ions. Figure 5b shows the illustrative and experimental shape transformation of folding and unfolding behavior of printed tube under the absence and presence of Ca^2+^ ions, respectively. They envisioned that the reversible shape change showed potential for capture and release of drugs and cells.

Lin et al. synthesized polypseudorotaxane hydrogels from *α*‐cyclodextrins (*α*‐CD) and Pluronic F127 (a commercial PEO–PPO–PEO triblock copolymer), and realized 3D printing of mechanically interlocked molecules.^[^ ^95^ ^]^ They investigated the influence of molar ratios of repeating units in copolymer to *α*‐CD on mechanical, rheological, and self‐healing properties for optimization. The introduction of free *α*‐CD facilitated the formation of hydrogen bonding network followed by photoirradiation to form polyrotaxane‐based monoliths with inter‐ring hydrogen‐bonding interactions (Figure 5c). When immersed the hydrogel in dimethyl sulfoxide (DMSO), the hydrogen‐bonding interactions was destroyed by solvent exchange, which enabled the a‐CD rings to shuttle along the axles of the polyrotaxanes and triggered macroscopic shape change. The reformation of hydrogen‐bonding interactions when immersed in water caused shape recovery up to five times. Similarly, Wang et al. printed SMH using sodium alginate and F127 diacrylate macromer (F127DA), which formed dual network internal structure.^[^ ^96^ ^]^ They first optimized the loading concentration of the ink components for the best quality of 3D printing. After UV crosslinking, the first crosslinking network was established as F127DA reacted with photoinitiator. Then, the printed structure was immersed in the Ca^2+^ solvent and formed the other crosslinking network to fix the temporary shape. Wang et al. evaluated the shape recovery process of printed scaffold under the removal of Ca^2+^ coordination stimulus within 30 min while about 90% recovery took in the first 10 min. Mulakkal et al. developed hydrogel composite ink containing a high content of cellulose fibers immersed in water to construct hydration‐ and dehydration‐responsive 4D printed structures based on structural design.^[^ ^97^ ^]^ The basic matrix was chosen as sodium carboxymethyl cellulose (CMC) polymer due to its biodegradable and cost‐efficient properties. The addition of pulp fibers and montmorillonite clay served to enhance the smooth printability and mechanical properties, respectively. A low content of citric acid was also added as a crosslinking agent to maximally fix the structures after 3D printing at low temperature. The shape transformation of a 4D hydrogel petal lied in the predefined strain mismatch which produced different strain upon hydration and dehydration, which mimicked the open and closure behavior of flowers.

#### 2.3.2 Thermal‐Responsive Hydrogels

Thermal‐responsive SMHs use temperature‐sensitive physical effects as reversible crosslinking points. Such physical effects include crystals, hydrogen bonds, and biological macromolecules with reversible structures. Bakarich et al. created a high‐toughness hydrogel with rapid and reversible shape change in water environment at around 32–35 °C.^[^ ^98^ ^]^ The thermal‐responsive poly(*N*‐isopropylacrylamide) network served as a toughening agent and also actuated volume transition for shape change. The 4D printed structure exhibited a large reversible strain of 41–49% and a blocking stress of 10–21 kPa. Subsequently, they printed an intelligent valve to control the flow rate of water. Similar to this method, they further printed alginate/poly(acrylamide) composite hydrogel ink into a valve, which exhibited different degrees of swelling to control the open and closure of valve at different water temperatures (Figure 5d).^[^ ^99^ ^]^ Lei et al. fabricated a 3D multifunctional skin‐like sensor which incorporated thermal‐responsive hydrogels into a capacitor circuit in a microstructured configuration, as illustrated in Figure 5e.^[^ ^100^ ^]^ The dual hydrogel networks were formed based on micellar‐copolymerization reaction, where physically crosslinked crystal domains imbedded in the covalent network. They found the 3D microstructures facilitated effective contacting area in the capacitor, thus increasing the sensitivity upon heating above volume phase transition temperature at 30 °C. As the temperature increased, the hydrogel became more transparent and the area gradually expanded. They further attached the sensor on skin and investigated the mutual influence of pressure and temperature on capacitance to monitor finger bending behavior.

Naficy et al. 4D printed reversible thermal‐responsive hydrogel structures based on long polymer chains and UV curable monomers.^[^ ^101^ ^]^ They fabricated hydrogel hinges which were consisted of bilayers made from *N*‐isopropylacrylamide and 2‐hydroxyethyl methacrylate (HEMA) hydrogel inks and investigated bending behavior upon thermal stimulus. Based on structural analysis, they designed a hydrogel cubic box with bilayers which self‐folded and opened at hydrated state at 20 and 60 °C, respectively, as shown in Figure 5f. Guo et al. demonstrated a 4D printed robust hydrogel consisting of agarose precursors, laponite and acrylamide through in situ polymerization of acrylamide.^[^ ^102^ ^]^ The 3D printed structure was post‐crosslinked to form two networks with hydrogen and chemical bonds, and the thermally sol–gel transition of agarose contributed to the fourth dimension with time. Figure 5g demonstrates a printed whale‐like structure opened its mouth and swung its tail, which seemed to come alive when heated at 95 °C by presetting deformation at first. The biological test proved that the printed hydrogel was nontoxic and the cell viability was above 90% in the first 3 days. Uchida and Onoe proposed a simple method to construct 4D thermal‐responsive multihydrogel structures by DIW printing in viscous liquid environment.^[^ ^103^ ^]^ The multilayer structure consisted of *N*‐isopropylacrylamide‐ and acrylamide‐based hydrogel by printing them in branched channels, while the former was thermal‐responsive but the latter was not. When the bilayer structure was heated, the thermal‐responsive part was sensitive and shrank. Figure 5h shows a representative shape‐morphing process of a C‐shaped structure transformed into a 3D spring at 48 °C.

#### 2.3.3 Photoresponsive Hydrogels

**Figure 6:**
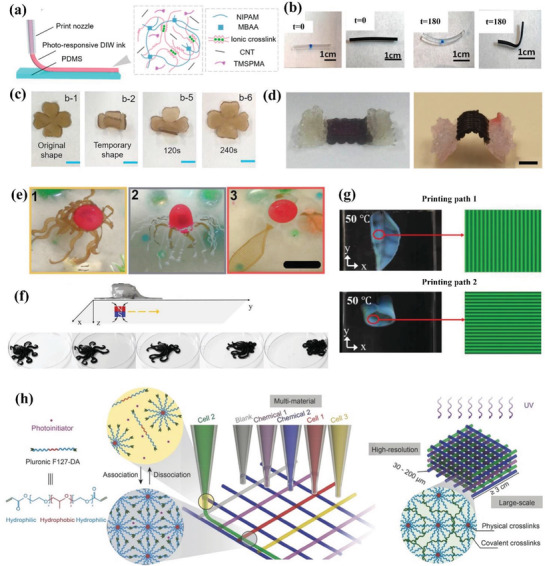
4D printing of photo‐ and other responsive hydrogels via DIW. a) Schematic diagram of 3D printing of poly(*N*‐isopropylacrylamide)‐based hydrogels/MWCNTs on PDMS and the composition of composite hydrogel ink. Reproduced with permission.^[^ ^104^ ^]^ Copyright 2019, American Chemical Society. b) Comparison of diameter shrinkage in photoresponsive hydrogel and hydrogel composite upon NIR irradiation. Reproduced with permission.^[^ ^105^ ^]^ Copyright 2017, American Chemical Society. c) Photoresponsive shape change of a printed flower consisting of F127DA/PLGA/GO hydrogel composite within 240 s (scale bars: 5 mm). Reproduced with permission.^[^ ^107^ ^]^ Copyright 2019, Elsevier. d) The saddle‐like shape change of a biphasic structure with different heights consisting of alginate and alginate/PDA composite (scale bar: 5 mm). Reproduced with permission.^[^ ^108^ ^]^ Copyright 2019, IOP Publishing. e) Magnetic‐responsive shape change of a printed seajelly‐like structure (scale bar: 15 mm). Reproduced with permission.^[^ ^109^ ^]^ Copyright 2019, Wiley‐VCH. f) Shape transformation of multistimuli‐responsive hydrogels in the shape of a printed octopus under a magnetic field, and g) different shape changes of printed leaves with different printing paths under heat stimulus. f,g) Reproduced with permission.^[^ ^112^ ^]^ Copyright 2019, Wiley‐VCH. h) Schematic diagram of printing programmed cells and chemicals within a scaffold. Reproduced with permission.^[^ ^114^ ^]^ Copyright 2018, Wiley‐VCH.

Unlike water‐ and thermal‐responsive hydrogels, photoresponsive hydrogels have no physical contact with the external environment during shape change, which can be actuated remotely. Cheng et al. prepared three types of hydrogels mixture and realized DIW printing by adding biocompatible alginate to regulate the rheological properties of hydrogel inks.^[^ ^104^ ^]^ They investigated the influence of content of alginate on viscosity, storage, and loss moduli of the ink, endowing the water‐like hydrogels precursor to possess shear‐thinning performance. By choosing different types of polymer precursors, they printed both chemically (acrylamide‐, acrylic acid‐, and *N*‐isopropylacrylamide‐based) and physically (poly‐vinyl, alcohol‐, and gelation‐based) crosslinking hydrogels. They further prepared photoresponsive ink consisting of *N*‐isopropylacrylamide‐based hydrogels and multi‐walled carbon nanotube (MWCNT). The photoresponsive hydrogel ink was directly printed on PDMS with modified interface and the schematic of 3D printing process and composition of ink is depicted in **Figure** **6**a. The excellent interface between hydrogel and PDMS layer was attributed to the silane coupling agent.

Basu et al. proposed a strategy to fabricate hydrogel composites by gel‐in‐gel printing through catalytically activated polymerization to trigger crosslinking.^[^ ^105^ ^]^ The MWCNT endowed the hydrogel with NIR photothermal response. The diameter shrinkage of printed hydrogel composite upon NIR laser was much larger than that in hydrogel as depicted in Figure 6b. Subsequently, they prepared a F127‐dimethacrylate‐based multistimuli‐responsive hydrogel ink to produce 3D structures containing yeast cells. The reversible stimuli‐responsive characteristics of hydrogel facilitated stable ink extrusion under appropriate temperature and pressure.^[^ ^106^ ^]^ The 3D printed lattice structure realized biocatalytic conversion of glucose into ethanol continuously. Dai et al. prepared SMH ink consisting of F127DA, polylactide‐*co*‐glycolide (PLGA), and graphene oxide (GO) to construct shape‐morphing structures under NIR irradiation.^[^ ^107^ ^]^ They found the addition of GO and PLGA enhanced shape memory and mechanical properties, respectively. The cell culture test on printed structure indicated no cytotoxicity and the shape recovery temperature triggered by NIR laser was close to body temperature which was safe. Figure 6c shows a representative shape‐changing process of printed composites within 240 s. Luo et al. prepared alginate‐based inks to construct cell‐laden structures with shape changing properties under NIR stimulus.^[^ ^108^ ^]^ The bilayer structures with orthogonal pattern printed by alginate/polydopamine (PDA) ink exhibited self‐folding behavior under NIR laser. They also designed biphasic structures with different heights made of alginate and alginate/PDA inks to transform into a saddle‐like shape as shown in Figure 6d.

#### 2.3.4 Other Stimuli‐Responsive Hydrogels

McCracken et al. developed 4D ionotropic hydrogels with tunable mechanical properties in hierarchical directions using DIW.^[^ ^109^ ^]^ They designed and mimicked the structure of seajelly organisms through spatial programming the valency of the ion‐binding agents. The introduction of iron oxide nanoparticles endowed the printed structure with shape‐changing behavior under local magnetic field, as illustrated in Figure 6e. This method integrated gray‐scale programming and gradient machanics within 4D printed structures. Similar to photoresponsive hydrogels, magnetic‐responsive hydrogels could be actuated remotely, avoiding physical contact with the external environment. Zolfagharian et al. used polyelectrolyte hydrogels ink consisting of gelatin and chitosan to print soft actuators with reversible bending behavior upon electrical stimulus.^[^ ^110^ ^]^ They compared the actuation behavior of both printed and cast film actuators and proved the superiority of functionality and delicate structures of the former. They further utilized topology optimization to optimize the structures of soft actuators for achieving large bending deflection.^[^ ^111^ ^]^ The experimental results revealed that the bending displacement and bending rate of printed actuators after topology optimization were more effective than 3D printed uniformly structures.

While the above mentioned hydrogels respond to a single stimulus, multistimuli‐responsive hydrogels can simultaneously integrate advantages of various single‐responsive hydrogels, showing great potential in building intelligent materials and expanding their applications. Chen et al. printed various hydrogels such as double network, magnetic‐responsive, and thermal‐responsive hydrogels, containing Carbomer at a minimum content of 0.5% w/v printed in the air without special setup.^[^ ^112^ ^]^ Carbomer served as both rheology modifier and supporting frame material, as well as enhancing mechanical properties. Compared to previous hydrogels, they printed hydrogel with much less additives and much higher water content. Figure 6f,g depicts two representative shape‐changing process of a printed octopus and a leaf model under magnetic and thermal stimuli, respectively. The moving forward motion of octopus showed promise in soft robotics. Lin et al. further synthesized polypseudorotaxane‐based hydrogels based on dimethacrylamide‐poly(ethylene‐glycol) and *α*‐CD, which served as axel and ring elements within the structure, respectively.^[^ ^113^ ^]^ After printing and post‐crosslinking, the modified polypseudorotaxane‐based hydrogels exhibited rapid and two‐way shape change upon pH variation in liquid. Then they copolymerized it with acrylate monomers to obtain polyrotaxane‐*co*‐polyacrylate network in response to both pH and ionic strength. Time‐evolving hydrogels exhibit biological response over time. Liu et al. introduced various bacterial cells and signaling chemicals into hydrogel ink to endow the printed structures with time‐evolving properties.^[^ ^114^ ^]^ Pluronic F127 diacrylate micelles dispersed in water facilitated the mixture to print since it provided shear‐thinning performance. Different engineered bacterial cells interacted with corresponding signaling chemicals, thus producing specific fluorescence under secretion of chemicals over time. The printing procedure is illustrated in Figure 6h with programmed cells and chemicals in certain printing locations. Highly organized 3D multicellular biological systems can achieve cell competition, proliferation, and apoptosis. Liu et al. further demonstrated three examples of printed living structures and proposed their potential application to be utilized as logic gates, spatiotemporal patterning devices, and wearable components for chemical detection on human skin.

## 3 4D Printing of Other Types of Materials

**Figure 7:**
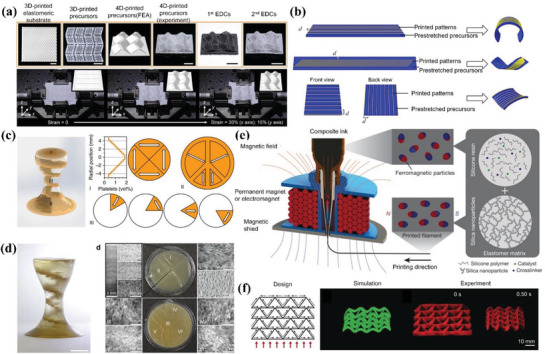
4D printing of elastomer‐derived ceramic and ferromagnetic domains via DIW. a) One method to construct 4D ceramics by printing Miura‐ori pattern and flexible joints on a prestretched elastomeric substrate followed by releasing external force to trigger bucking (scale bars: 1 cm). b) FEA analysis of the other method to construct 4D ceramics by designing the directional relationship between printed filaments and prestretched substrate. a,b) Reproduced with permission.^[^ ^119^ ^]^ Copyright 2018, American Association for the Advancement of Science. c) Structural design of multimaterial in both composition and location. d) Printed multimaterial and characterization of alignment of magnetized particles in targeted area (scale bar: 5 mm). c,d) Reproduced with permission.^[^ ^120^ ^]^ Copyright 2015, Nature Publishing Group. e) Schematic illustration of oriented ferromagnetic particles during printing under a magnetic field with programmed magnetic polarity. f) Shape transformation of auxetic behavior within 0.5 s under a magnetic field of 200 mT. e,f) Reproduced with permission.^[^ ^11^ ^]^ Copyright 2018, Nature Publishing Group.

Polymer‐derived ceramics (PDCs) have been investigated over 40 years.^[^ ^115^ ^]^ They are fabricated through thermolysis of preceramic polymers under inert atmosphere, which enables polymers to transform to ceramics. By regulating polymeric system and thermolysis conditions, PDCs have shown great potential in many fields due to their unique microstructures and properties such as chemical durability and creep resistance.^[^ ^116^ ^]^ The development of 3D printing enabled ceramic precursors to construct complicated structures and cellular architectures, which was hard by traditional methods.^[^ ^117^, ^118^ ^]^ Recently, Liu et al. proposed 4D printing of elastomer‐derived ceramics (EDCs) based on silicone rubber nanocomposites by DIW.^[^ ^119^ ^]^ The ink of ZrO_2_/PDMS was printed into 3D elastomeric lattices. The shape‐changing process was realized by deforming 3D printed elastomeric lattices into origami structures with the help of metal wire. Based on Gauss's theorema egregium, the precursors presented sophisticated architectures such as butterfly, the Sydney Opera House, rose, and dress. After thermolysis, the metal wire was dissolved in HNO_3_ and the complicated ceramics could be tuned between black and white using different heat treatment methods. The utilization of stretch device enabled 4D printing of EDCs. One method was to print precursor with Miura‐ori pattern and flexible joints on a prestretched elastomeric substrate. After releasing external force, the compressive force of the substrate caused bucking of the precursor architecture, as illustrated in **Figure** **7**a. The other method was to print parallel filaments on the prestretched precursors. Figure 7b shows finite element analysis (FEA) of three shape transformations such as bending, twisting and saddling by designing the directional relationship between printed patterns and prestretched substrate. They envisioned this light but strong ceramic structures based on shape‐changing characteristics of elastomers would pave the way for space applications.

The deliberate control of the alignment of magnetized particles during 3D printing was first achieved by Kokkinis et al.^[^ ^120^ ^]^ The ink mainly consisted of polyurethane acrylate (PUA) as matrix, HEMA as mechanical modifier, SiO_2_ as rheology modifier, and alumina platelets as oriented functional component. Assisted by a magnetic field and two‐component dispenser, the spatial programming of concentration and gradient in multimaterial composites is shown in Figure 7c. Based on this design, the printed multimaterial structure was observed in scanning electron microscopy (SEM) images to prove the accurate alignment of magnetized particles in specific area as demonstrated in Figure 7d. The working mechanism for shape change relied on different swelling strains of the stiff and soft parts in the multimaterial when immersed in ethyl acetate. Different from Liu et al.^[^ ^119^ ^]^ and other researchers^[^ ^121^, ^122^ ^]^ who realized transformation from 2D planar to 3D complex structures by buckling of materials on prestretched elastomer, Kim et al. enabled fast noncontact shape change between complex 3D shapes by magnetic field.^[^ ^11^ ^]^ Figure 7e shows programmed by a magnetic field during printing, the ferromagnetic particles (NdFeB) were reoriented in soft elastomer ink, which enabled the printed filament to possess patterned magnetic polarity. Based on the design of magnetic polarity and FEA, they remotely realized bending, auxetic, Miura‐ori, and more complex folding behavior with fast transformation under a magnetic field. One representative auxetic behavior illustrated in Figure 7f completed shape transformation within 0.5 s under a magnetic field of 200 mT. Both the actuation time (0.1–0.5 s) and power density (≈22–309 kW m^−3^) were orders of magnitude higher than reported 3D printed shape‐changing soft materials.

**Figure 8:**
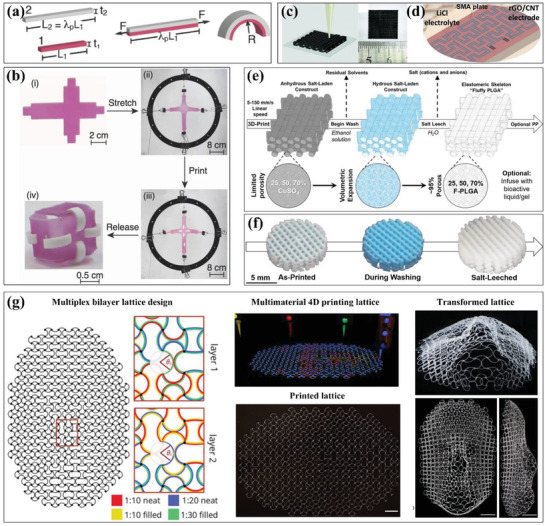
4D printing of other types of materials via DIW. a) Schematic mechanism of transformation from a 2D planar to 3D bending shape. b) Fabrication procedure to construct a cubic box in three steps. a,b) Reproduced with permission.^[^ ^123^ ^]^ Copyright 2019, Wiley‐VCH. c) Optical images of 3D printed VO_2_/PDMS THz photonic crystals with a woodpile structure. Reproduced with permission.^[^ ^124^ ^]^ Copyright 2019, Royal Society of Chemistry. d) Schematic diagram of thermal‐responsive supercapacitor consisting of SMA substrate, rGO/CNT electrode, and LiCL electrolyte. Reproduced with permission.^[^ ^125^ ^]^ Copyright 2019, Wiley‐VCH. e) Schematic graph of color and shape transformation of CuSO_4_/PLGA scaffold in response to ethanol and water, respectively. f) Transformation of printed circular scaffold after printing, washing, and salt‐leaching in sequence. e,f) Reproduced with permission.^[^ ^126^ ^]^ Copyright 2018, Elsevier. g) Multiplex bilayer lattice design consisting of four PDMS‐based materials, multimaterial 4D printing process, and printed lattice of a planar human face and the morphing curved shape under heat stimulus. Reproduced with permission.^[^ ^131^ ^]^ Copyright 2019, National Academy of Sciences.

Like Liu et al.,^[^ ^119^ ^]^ Cafferty et al. realized transformation from 2D planar to 3D complex structures based on prestretched elastomer and the principle is illustrated in **Figure** **8**a.^[^ ^123^ ^]^ They first prestretched the bottom layer on which the top layer was printed later and both the bilayers consisted of liquid silicones. After curing and removing the external force, the elastic energy stored in prestretched layer released and triggered the bending behavior of bilayer structure. By regulating the prestretched ratio, they optimized the bending curvature and obtained helix and hemihelix when the circumference of the bending circle was smaller than the original length. This method provided a simpler route to obtain complicated structures and suspended geometries with a faster printing speed in the 2D platform. Figure 8b shows the fabrication procedure to construct a cubic box in three steps: i) stretched the flat elastomeric substrate; ii) printed the elastomer ink on the substrate while remaining stretching; iii) released the structure and it self‐folded into a cubic box. Wang et al. prepared a composite ink composed of vanadium dioxide (VO_2_) nanoparticles/PDMS for DIW.^[^ ^124^ ^]^ By regulating the structural parameters such as spacing between adjacent filaments and layer thickness, they created 3D terahertz photonic crystals with woodpile architecture as shown in Figure 8c. Other than structural design of printing, they also found that the printed device showed changeable permittivity under thermal stimulus due to the introduction of VO_2_. The intrinsic phase transition of VO_2_ from insulator phase at room temperature to metallic phase at 75 °C caused dramatic changes of electrical properties, which endowed the nanocomposites with thermally tunable transmission properties.

Jiang et al. integrated reduced graphene oxide (rGO)/CNT electrodes and lithium chloride (LiCL) electrolyte into thermal‐responsive shape memory alloys to construct complex supercapacitor.^[^ ^125^ ^]^ The schematic architecture of DIW‐assisted integrated supercapacitor is shown in Figure 8d. They investigated the influence of printing parameters on printing quality and printed structures with high resolution as high as 175 µm, which was far beyond other reported carbon‐based storage devices. Jakus et al. combined 3D printing with salt‐leaching to fabricate high porous scaffolds, which extracted salt and increased the size.^[^ ^126^ ^]^ By controlling the volume ratio of CuSO_4_ salt to PLGA at medical grade, the mechanical, physical, porous, and biological properties were tailored. They proved the high porous scaffold containing 70 vol% salt endowed the adult human mesenchymal stem cell with good attachment and proliferation within a month. Figure 8e shows schematic graph of two steps including washing in ethanol and salt‐leaching in water solution, which caused color transformation and shape transformation, respectively. The blue color transformation visually indicated the hydratation of CuSO_4_ as it dissociated ions, but not entered the solution. The process of salt‐leaching formed large amount of porous and caused dimensional expansion in size. These two continuous transformations are visually demonstrated in Figure 8f. Mu et al. realized 4D printing of Ag NPs onto a printed elastomer.^[^ ^127^ ^]^ They investigated the influence of thermal cure condition (i.e., curing time and temperature) on conductivity and electromechanical properties to obtain both sensitive conductivity and large stretchability. They found that with the increase of curing temperature and time, the electrical conductivity of Ag NPs increased, while elongation at break decreased. They obtained a sandwich structure of Ag NPs embedded in an elastomer by a sequential printing method of DIW and PolyJet technique. They further realized intense pulsed light (IPL) sintering of thick Ag NPs onto a printed elastomer and investigated the sintering conditions on resistivity and electromechanical properties.^[^ ^128^ ^]^ They proved that the IPL sintering was a rapid and efficient method for conductive hybrid 4D printing. They believed the construction of conductive and stretchable devices with customer‐designed geometries would pave the way for applications in 4D printing. Deng et al. utilized a similar method to realize 4D printing of a self‐foldable electronic circuit.^[^ ^129^ ^]^ The thermal‐responsive shrinkable film in an integrated structure triggered the shape transformation of the DIW printed silver ink under heat stimulus.

Unlike the printed materials with shape‐changing capability, Su et al. proposed a method of 4D printing by incorporating a swellable guest medium into a nonswellable host polymer.^[^ ^130^ ^]^ They investigated the influence of key parameters such as thickness, length, and printed geometries on self‐morphing behavior, both from FEA and experiments. By spatial control of the active and passive patterns, the printed structures showed reversible shape change when immersed in acetone and water repeatedly. Since the utilization of printed PDMS to construct shape‐shifting structures were usually confined in simple architectures, Boley et al. proposed a new method to achieve complex and doubly curved shapes based on theoretical prediction.^[^ ^131^ ^]^ They utilized multiple materials with heterogeneous lattice designs to accurately control the metric tensor over both space and time. The four printable inks consisted of two PDMS‐based mixtures with various weight ratios of crosslinking agents and glass fibers, which regulated the modulus and thermal expansion coefficient. The printed lattice pattern had curved bilayer ribs to achieve in‐plane elongation or contraction and out‐of‐plane transformation under thermal stimulus. They also demonstrated the polymorphism when immersed the printed lattice in solvents by programming the Gaussian curvature. Then they controlled both the intrinsic and extrinsic curvatures by designing multiplex bilayer lattice to achieve complex transformation from plane to a 3D human face, as depicted in Figure 8g. Compared to previous work,^[^ ^92^, ^132^ ^]^ their new methods had two advantages. One was the lattice had high tunability of local linear in‐plane growth which enabled the lattice at various scales. The other was the out‐of‐plane bending control reduced elastic frustration and simplified inverse design of complex structures.

## 4 4D Printing of Functional Materials

The above mentioned DIW‐based 4D printing materials is classified from the types of materials. In this section, we will emphasis on their multifunctional properties, especially self‐healing properties. Self‐healing property refers to the ability that structures and functions are capable of recovering after damages so that their material reliability and lifetime increase.^[^ ^133^, ^134^, ^135^, ^136^ ^]^

**Figure 9:**
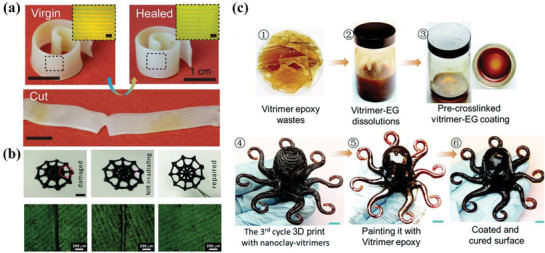
4D printing via DIW for self‐healing functionality. a) Shape memory assisted self‐healing process of a damaged spiral by heating above the melting temperature of PCL. Reproduced with permission.^[^ ^65^ ^]^ Copyright 2018, American Chemical Society. b) In situ self‐healing of a printed spider‐like structure under NIR irradiation at damaged location and the enlarged optical image of damaged crack (scale bar: 1 cm). Reproduced with permission.^[^ ^66^ ^]^ Copyright 2019, Royal Society of Chemistry. c) Recycling process of vitrimer epoxy and the comparison of a printed octopus after recycling and self‐healing (scale bar: 8 mm). Reproduced with permission.^[^ ^137^ ^]^ Copyright 2017, Royal Society of Chemistry.

Kuang et al. endowed the 4D printed structures based on SMP with self‐healing capabilities.^[^ ^65^ ^]^ After printing, the spiral was cut by a blade and deformed into a strip as temporary shape as shown in **Figure** **9**a. Once heated above the melting temperature of PCL, the crystals melted and entangled, thereby closing the crack and recovering to its original shape. The self‐healing process was also verified by three damage‐healing cycles to prove the healing efficacy. Based on the successful synthesis of PDAPU with photothermal effect, Zhang et al. proved its light‐actuated self‐healing capabilities.^[^ ^66^ ^]^ When the edges of a printed spider‐like structure were broken, NIR irradiation facilitated the crack closure in situ, as shown in Figure 9b. The fractured part was closed within 10 s under a NIR power of 0.8 W, exhibiting high self‐healing efficiency. Once reached the thermal transition temperature of soft segment in PDAPU, shape recovery could occur to help restore its original shape. Compared to direct heating, in situ NIR irradiation in fractured area had little impact on other areas. The self‐healing efficiency reached above 70% after three facture‐healing cycles. Shi et al. 4D printed thermosetting polymers which could be recycled four times for next cycle of printing, while retaining good stability and repeatability.^[^ ^137^ ^]^ The printed vitrimer epoxy was thermoset at room temperature but the networks could be converted by the transesterification type bond exchange reaction (BER) and further dissolved in ethylene glycol (EG) upon heating.^[^ ^138^, ^139^ ^]^ Then, Shi et al. matched the vitrimer ink with DIW setup by modulating the ink viscosity, heating temperature and pre‐crosslinking time to construct complex geometries. The healing mechanism was solvent‐assisted surface welding, which not only repaired the cracks but also improved the surface gloss. The recycling procedure of vitrimer epoxy and the comparison of a printed octopus after recycling and self‐healing are illustrated in Figure 9c.

In addition to self‐healing properties, DIW‐based 4D printed structures also presented other functionalities, such as optics,^[^ ^82^, ^124^ ^]^ conductivity,^[^ ^60^ ^]^ biological response,^[^ ^114^ ^]^ and color transformation^[^ ^126^ ^]^ along with the process of shape change. We could see these works explored various functionalities which enabled the printed structures to play a greater role in 4D printing area.

## 5 4D Printing for Various Applications

### 5.1 Biomedical Field

The tunable biodegradability of 4D printed materials alongside their time‐evolving properties make them candidate materials applied in biomedical field. In this area, some important parameters including biocompatibility, actuation temperature, and shape‐changing rate are usually paid attention on.

**Figure 10:**
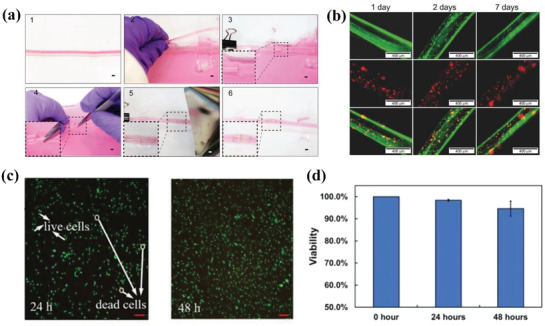
4D printing via DIW for biomedical applications. a) Programming and shape recovering of a printed hollow tube to simulate vascular repair (scale bars: 2 mm). Reproduced with permission.^[^ ^65^ ^]^ Copyright 2019, American Chemical Society. b) Fluorescence microscopy images of the printed cell‐laden hydrogel tubes during cell culture in 7 days. The first, second, and third rows corresponded to live, dead, and all cells in the printed structure, respectively. Reproduced with permission.^[^ ^94^ ^]^ Copyright 2019, Wiley‐VCH. c) Fluorescence microscopy images of the printed PEGDA/carbomer hydrogel during cell culture within 48 h (scale bars: 100 µm). d) Calculated cell viability of PEGDA/carbomer hydrogel. c,d) Reproduced with permission.^[^ ^112^ ^]^ Copyright 2019, Wiley‐VCH.

The self‐expanding behavior of 4D printed SMPs and SMPCs showed the possibility as vascular stents to support blood vessel.^[^ ^16^, ^58^ ^]^ Similarly, Wang et al. performed a defect filling experiment in vitro to demonstrate the possibility of implantation in minimally invasive surgery.^[^ ^61^ ^]^ The original scaffold was compressed into a small temporary shape with the volume shrinkage of 44%. When heated in 37 °C water bath, the scaffold showed self‐expanding behavior in 180 s with shape recovery ratio of ≈94%. After obtaining shape memory assisted self‐healing properties, Kuang et al. further showed an in vitro example that semi‐IPN elastomer exhibited application for vascular repair.^[^ ^65^ ^]^ They used silicone tube filled with red fluid to simulate the environment of a blood vessel. After breaking the silicone into two pieces, two edges of the silicone tube were clamped to simulate hemostasis. The printed hollow tube was stretched into a small diameter shape, which was much smaller than the inner diameter of silicone tube to facilitate implantation into the crack. Under heat stimulus, the deformed tube recovered to its initial shape within 30 s, showing a self‐expanding behavior to fully contact with the inner of silicone tube as depicted in **Figure** **10**a. Thus, it showed great potential for reconnecting and repairing the broken “blood vessel” by a facile method.

Although the shape change of high porous scaffold did not serve directly, Jakus et al. found the microstructures of printed scaffold resembled that of tissue or organ derived extracellular matrices.^[^ ^126^ ^]^ The modulus range was similar to auricular cartilage and the scaffold could serve as a carrier to implant and transport weak biomaterials such as gels in a targeted location. After printing shape‐morphing polymer‐cell hollow structures, Kirillova et al. further investigated the differentiation of various cell types under solvent and found it had no effect on the cell viability within 7 days (Figure 10b).^[^ ^94^ ^]^ Thus, the 4D dynamically reconfigurable constructs with tunable functionality and responsiveness opened new exports of creating tailored cell‐laden shape‐morphing architectures for tissue engineering and regenerative medicine applications. Wang et al. extended the application of printed SMH into drug delivery applications by loading an anticancer drug, methotrexate.^[^ ^96^ ^]^ They compared the in vitro drug release results and found the printed SMH released more drug in the same period than the traditional manufactured SMH. This was because the internal grids fabricated by 3D printing increased the surface area of the drugs. Besides, the presence of alginate as a type of polysaccharides could support nutrients for printed structure to promote cell growth. The excellent biocompatibility and slight degradation showed the potential of 4D printed SMH as a short‐term implantable device for local drug release. The catalytically active living materials fabricated by Saha et al. exhibited excellent viability and metabolic activity of the yeast cells in the hydrogel.^[^ ^106^ ^]^ They believed that by optimizing microbial species or cell types during cell culture or incorporating microbes with engineered genetic modifications, the smart hydrogel was valuable for antibiotics and drug therapeutics. The 4D cell‐laden structures constructed by Luo et al. retained high cell viability for at least two weeks and they found the NIR laser irradiation had no obvious influence on laden cells.^[^ ^108^ ^]^ Thus they believed the printed shape‐morphing structures with bioactivity may be used in tissue engineering and regenerative medicine, which required bending curvatures. By modifying hydrogel inks with carbomer, Chen et al. found the printed material was cytocompatible, thus showing potential in biomedical field.^[^ ^112^ ^]^ They chose printed poly(ethylene glycol) diacrylate (PEGDA)‐carbomer hydrogel to analyze the biocompatibility within 48 h and the result showed excellent cell viability as shown in Figure 10c,d.

### 5.2 Electronics

The electrical response of 4D printed materials enables them to be applied in the field of electronics. In this area, some important characteristics such as actuation voltage, electrical conductivity, mechanical properties, and shape‐shifting rate are usually considered.

**Figure 11:**
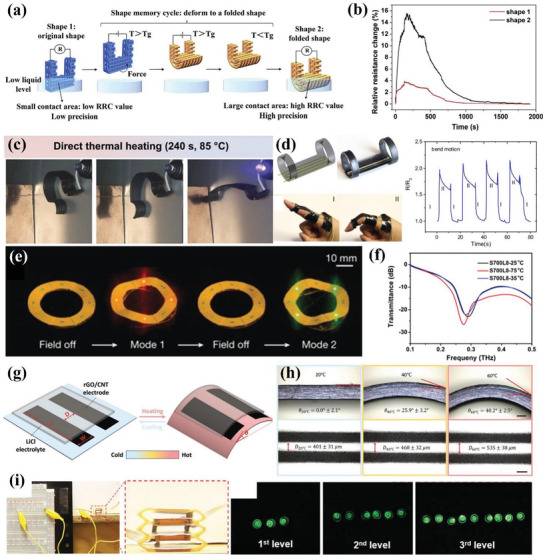
4D printing via DIW for electronics. a) Schematic illustration of a shape‐changing sensor with high detecting precision when the liquid level decreased. b) Comparison of relative resistance change between flat and folded sensors immersed in ethanol. a,b) Reproduced with permission.^[^ ^60^ ^]^ Copyright 2019, Elsevier. c) Printed electrical component stretched upon heating at 85 °C and lighted up a LED light. Reproduced with permission.^[^ ^10^ ^]^ Copyright 2016, Nature Publishing Group. d) Printed Ag NPs/elastomer as a flex sensor to detect the motion of finger bending and the relative resistance change during four cycles. Reproduced with permission.^[^ ^127^ ^]^ Copyright 2017, IOP Publishing. e) Programming of printed ferromagnetic domains in response to different magnetic directions to trigger different circuits. Reproduced with permission.^[^ ^11^ ^]^ Copyright 2018, Nature Publishing Group. f) THz transmittance spectra of 3D photonic crystals at different temperatures. Reproduced with permission.^[^ ^124^ ^]^ Copyright 2019, Royal Society of Chemistry. g,h) Schematic and experimental shape change with a bending angle of the printed supercapacitor upon heat stimulus. Reproduced with permission.^[^ ^125^ ^]^ Copyright 2019, Wiley‐VCH. i) The printed multilevel triboelectric nanogenerator was used as an energy harvester to supply power for LEDs under sequential compressive modes. Reproduced with permission.^[^ ^68^ ^]^ Copyright 2019, Wiley‐VCH.

After improving the gel fraction and mechanical strength of conductive nanocomposites, Wan et al. printed electroresponsive shape‐changing liquid sensors to detect various liquids.^[^ ^60^ ^]^ They found that the shape‐changing capability helped the sensor adapt to varying environments and maintain high detecting precision simultaneously. As shown in **Figure** **11**a, when the liquid level of detecting environment decreased, the flat sensor was deformed into a folded shape. Since the new shape had larger contact area between the sensor and liquid, it exhibited a relative resistance change almost four times that of a flat sensor when immersed in ethanol, as shown in Figure 11b. This new type of shape‐changing sensor showed great potential for environmental adaptability and also saved cost of materials. Rodriguez et al. printed an electro‐cresponsive 4D SMPC structure with CNF/ESBO/BFDGE ink.^[^ ^10^ ^]^ The deformed structure was placed in the vicinity to a copper electrode, leaving an open circuit in a 9 V electric field. Figure 11c shows when placed the whole circuit in 85 °C, the electrical structure showed stretchable shape‐changing behavior, which closed the circuit and powered an LED light. Afterward, they connected this electrical structure into a 20 V power and tested the surface temperature over time. They found that upon resistively heating to higher temperature, the shape‐changing rates increased and triggered full shape recovery. This research proves that complex conductive structures can be designed and fabricated by 4D printing, showing the potential as low‐power electrical components. Mu et al. further showed two applications by printing Ag NPs onto 3D printed elastomer.^[^ ^127^ ^]^ One was the sandwich structure which could be connected into a circuit as an electrical component. When the sandwich structure was stretched by 45%, it still retained high conductivity to turn on a LED light at ≈500 mW. Figure 11d shows the other potential application as a flex sensor. By printing Ag NPs onto the bottom side of elastomer, the resistance of the structure would increase upon stretching. Thus, it could be utilized to detect the motion of finger bending.

Based on the programming of ferromagnetic domains within the printed structure, Kim et al. demonstrated an electronic device with different electronic functions in response to varying directions of an applied magnetic field as shown in Figure 11e.^[^ ^11^ ^]^ They integrated electronic components and circuitry with a printed annular structure with programmed magnetic polarities, and the different shape change under different magnetic directions triggered different circuits, thus lighting up LED lights with different colors. It showed potential to fabricate reconfigurable complex electronic devices with fast, remotely actuated behavior. The 3D photonic crystals printed by Wang et al. showed increasing bandgaps in the terahertz frequency range when the temperature was higher than the insulator–metal transition temperature.^[^ ^124^ ^]^ It exhibited large transmission decay at a maximum value at 44% and excellent reversibility when the temperature returned to about room temperature, as shown in Figure 11f. The reversibly thermal‐responsive 3D photonic crystals with remotely tunable permittivity showed great potential for integrated terahertz devices. Su et al. displayed the shape transformation of self‐morphing polymer as a soft electronic relay to close a circuit.^[^ ^130^ ^]^ When immersed in acetone, the original flat shape started to bend which made the conductive wires connected and lighted up a LED light. This printed self‐morphing soft actuator showed superiority of simple fabrication of an electrical relay. The integrated supercapacitor fabricated by Jiang et al. possessed excellent thermal‐responsive behavior of SMA, which exhibited dynamic charge and discharge rate capability at different temperatures.^[^ ^125^ ^]^ Figure 11g,h schematically and experimentally illustrate the reversible shape change of supercapacitor under heat stimulus, respectively. As the temperature increased, the device bended thus the distance between two electrodes increased to prevent thermal runaway. Compared with SMP, the two‐way actuation behavior of SMA made it more reliable to repeat reversibly thousands of bending cycles. Besides, the specific capacitance of the printed device was as high as 8 F g^−1^, which helped the device protect against thermal runaway. Such self‐protect capability alleviated serous safety problems for wearable applications.

After printing graded three‐layer multimaterial through photomask‐assisted DIW, Chen et al. designed a multilevel triboelectric nanogenerator as a self‐powered wearable device.^[^ ^68^ ^]^ The three graded layers had different stiffness, thus exhibiting sequential deformation under mechanical forces. It was used as an energy harvester to enlighten different numbers of LEDs when different layers were compressed under external force, as shown in Figure 11i. After printing multimaterial combined with eutectic gallium indium ink into planar lattices, Boley et al. used the shape‐shifting structures as a hemispherical antenna which changed resonant frequency at various temperatures.^[^ ^131^ ^]^ The printed structure morphed into a spherical cap when temperature decreased. The fundamental resonant frequency decreased as the height of cap increased which showed potential for wireless sensing and dynamic communication. Considering the shape‐shifting antennas were conductive and had high toughness, they were promising in soft electronics.

### 5.3 Soft Robotics and Actuators

Another important application of 4D printed materials is the area of soft robotics and actuators. In this field, the overall properties of actuation stress, work capacity and repeatability are commonly concerned.

**Figure 12:**
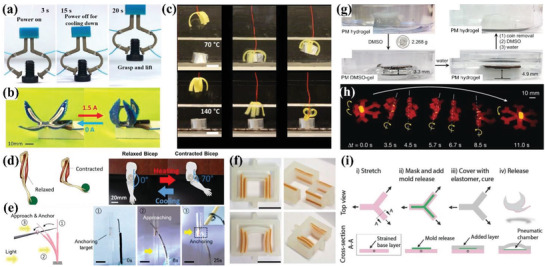
4D printing via DIW for soft robotics and actuators. a) The shape change of a printed SMPC gripper lifting a bolt under a 1 V voltage. Reproduced with permission.^[^ ^56^ ^]^ Copyright 2019, American Chemical Society. b) Two‐way shape change of a LCE gripper when the electric current was on and off. Reproduced with permission.^[^ ^83^ ^]^ Copyright 2018, IOP Publishing. c) Grabbing behavior at low temperature and not grabbing behavior at high temperature by programming LCEs with different *T*_NI_s with different printing directions (scale bars: 10 mm). Reproduced with permission.^[^ ^84^ ^]^ Copyright 2019, Wiley‐VCH. d) Twining printed LCE fibers together to mimic the bending motion of bicep muscle fibers to grab objects upon heat stimulus. Reproduced with permission.^[^ ^85^ ^]^ Copyright 2019, American Chemical Society. e) Biomimetic bending and anchoring behavior of an artificial tendril consisting of photoresponsive hydrogel/PDMS. The light source moved from the base part to tip part of the structure successively to actuate the shape change (scale bars: 10 mm). Reproduced with permission.^[^ ^104^ ^]^ Copyright 2019, American Chemical Society. f) Mechanical fastening behavior of magnetic‐assisted multimaterials upon liquid immersion (top: as‐printed valve; bottom: immersed in ethyl acetate. Scale bars: 15 mm). Reproduced with permission.^[^ ^120^ ^]^ Copyright 2015, Nature Publishing Group. g) Printed water‐responsive hydrogel converted chemical energy to mechanical work, lifting a coin upward. Reproduced with permission.^[^ ^95^ ^]^ Copyright 2017, Wiley‐VCH. h) Printed ferromagnetic domains carried and released a pill under a rotating magnetic field. Reproduced with permission.^[^ ^11^ ^]^ Copyright 2018, Nature Publishing Group. i) Mechanically and pneumatically actuated gripper based on prestretched 2D elastomers. Reproduced with permission.^[^ ^123^ ^]^ Copyright 2019, Wiley‐VCH.

Wei et al. demonstrated the application of printing PLA/Ag@CNF nanocomposite into smart grippers.^[^ ^56^ ^]^ **Figure** **12**a shows the electro‐responsive shape‐changing behavior of a printed gripper containing 10.6 vol% Ag@CNF within 20 s under a voltage of 1 V, which grabbed a bolt. The low voltage to trigger shape memory behavior exhibits great advantages when the applied voltage is limited, thus saving energy. Although they did not test the recovery stress during shape actuation directly, this mechanical performance of 4D printed PLA‐based SMPC structures could be found in similar literature. Senatov et al. reported that a 4D printed PLA/hydroxyapatite porous scaffold actuated a recovery stress of ≈2.8 MPa.^[^ ^140^ ^]^ Zolfagharian et al. also illustrated the potential application of SMPC as soft actuators.^[^ ^62^ ^]^ They printed an artificial hand with three fingers, which exhibited bending behavior under NIR irradiation. By regulating the variables, they achieved sequential bending behavior with designed bending angle and bending speed. The produced blocking stress and work capacity upon nematic–isotropic transition enabled 4D printed LCEs to be used in soft robotics and actuators. On the basis of reversible shape change assisted by LCE and hybrid printing, Yuan et al. fabricated smart actuators.^[^ ^79^ ^]^ By designing two sets of silver wire at different locations, they achieved sequential folding of a box. They used two materials with different friction coefficients on the two ends of the laminated structure and mimicked the locomotion behavior under a current signal. Based on the combination of opposing Gaussian curvatures with topological alignment, Ambulo et al. endowed the printed LCE with rapid and repetitive snap‐through deformations.^[^ ^80^ ^]^ The snap‐through actuation could lift a load five times heavier than the LCE actuator. Under loads less than 20 times the weight of the LCE actuator, the snap‐through actuation performed specific work in the range of 0.1–0.7 J kg^−1^. Using the same materials system but with a decreased molar ratio of RM 82:*n*‐butylamine, Kotikian et al.^[^ ^81^ ^]^ 4D printed LCE actuators with large and two‐way transformation. The 4D printed actuator could lift loads with a maximum value of ≈106 mg, 1000 times heavier than itself upon heating above *T*_NI_, much higher than previous literature. As the weight of load increased, the actuation strain of the LCE actuator decreased while the specific work capacity increased up to 39 J kg^−1^. The work capacity of 4D printed LCE actuators was significantly increased by such a simple method. A moderate molar ratio of RM 82:*n*‐butylamine in the range of these two groups^[^ ^75^, ^76^ ^]^ resulted in a moderate work capacity of 1.44 J kg^−1^.^[^ ^77^ ^]^

Roach et al. utilized the hybrid printing of LCE, soft substrate and conductive wires to create a soft griper, as shown in Figure 12b, which picked up and placed a ball when current was on or off.^[^ ^83^ ^]^ The hinges consisted of four LCE strips, which showed bending behavior under the Joule effect of conductive wires. They further fabricated a robotic hand mimicking the bending of every finger where different locations were electrically heated. By incorporating multimaterial within a LCE structure, Saed et al. demonstrated sequential shape transformation of a temperature sensitive gripper, which was different from the previous literatures.^[^ ^84^ ^]^ By assigning LCE with different *T*_NI_s on two sides of the structure and designing different printing directions between layers within a single structure, they obtained two bending behavior with opposite curvatures in a sequential heating process. Figure 12c illustrates the low‐temperature grabbing and high‐temperature not grabbing behavior. The intrinsic properties of printed multi‐LCE provided major implications and advances in the field of soft robotics. By twining the printed LCE fibers together, Roach et al. simulated the bending behavior of bicep muscle fibers to grab loads.^[^ ^85^ ^]^ Figure 12d shows the lifting motion between relaxed and contracted status, where LCE fibers were located at the position near the elbow. When heated, the LCE fiber contracted thus the arm bended to mimic the motion of bicep muscle. It also revealed that the actuation stress increased as the number of twined fibers increased, with a maximum lifting weight 250 times heavier than own. Considering the temperature difference existed along the thickness of printed LCE filament, Zhang et al. printed a LCE strip which lifted an object ≈600 times its own weight with a lifting height of 7 mm.^[^ ^87^ ^]^ Upon heating, the LCE actuator generated a maximum actuation stress of 0.31 MPa. By controlling the areas with temperature gradient or not, they further designed two deformation patterns and presented their potential applications as soft actuators under heat stimulus.

Compared to LCEs, hydrogels exhibited a slightly higher blocking stress (0.01–2 MPa) and work capacity during shape transformation, especially for water‐responsive hydrogels.^[^ ^91^, ^98^ ^]^ After preparing photoresponsive poly(*N*‐isopropylacrylamide)/MWCNT composite hydrogel ink, Cheng et al. designed complex structures consisting of composite hydrogel and PDMS to mimic biomimetic bending and anchoring behavior of natural plant tendril.^[^ ^104^ ^]^ When light produced by a xenon lamp came from different directions, photothermal MWCNT within the artificial tendril bended toward the light source. Figure 12e shows successive shape change of the artificial tendril upon light irradiation. When light illuminated base part, the structure started to bend and approach toward a fixed target, followed by anchoring to it as light source moved to irradiate the tip part. They also 4D printed an artificial tentacle with an actuation force of ≈0.45 N. This research expanded the functionalities of hydrogels to soft robotics with phototropic motion.

Figure 12f illustrates the mechanical fastening behavior of magnetic‐assisted multimaterials performed by Kokkinis et al.^[^ ^120^ ^]^ The smart shape‐changing fastener with complementary structures behaved as smart key–lock connectors, which generated bending behavior upon swelling in ethyl acetate. The fastening mechanism was the bilayer walls of the key and lock formed concave and convex curvature, respectively. The reconfigurable fastener with key–lock shape change lifted an object much heavier than its own weight, because it combined both the frictional force and swelling stress. After synthesizing polypseudorotaxane hydrogels, Lin et al. further utilized the printed hydrogels as smart actuators.^[^ ^95^ ^]^ Figure 12g shows the chemical energy of water‐responsive hydrogel converted to mechanical work in a solvent cycle, which produced 30.5 µJ of work and lifted a metal coin upward 1.6 mm. Kim et al. demonstrated fast shape transformation of a printed hexapedal with programmed magnetic polarity within the structure, which could stop and hold an object with fast moving velocity by a magnet.^[^ ^11^ ^]^ Figure 12h shows by applying a rotating magnetic field, the structure wrapped, carried, and released an oblong pharmaceutical pill when exposed at different directions of magnet. It revealed the possibility of controlling the locomotion of printed soft devices at desired locations with desired shapes. Cafferty et al. fabricated grippers by combining printed 2D elastomers with release of strain, as illustrated in Figure 12i.^[^ ^123^ ^]^ They added the mask on the prestrained elastomer to control the exposed and unexposed surface according to whether the top ink needed to be adhered. Different from previous grippers, they preset pneumatic chamber by using mold release, which was located between the strained and printed layers. The modified chamber enabled a tube to be inserted to inflate the structure and realized both the mechanically and pneumatically actuation method. McCracken et al. fabricated soft aquatic actuators whose shape transformed into biomimetic 4D structures under hydration stimulus.^[^ ^109^ ^]^ The gradient and programmed mechanics within the structures actuated the shape change which were both determined by magnetic field and preset shape. They proposed a cutting‐edge method to print 4D complicated dynamically responsive biomimetic structures.

## 6 Summary of Achievements, Challenges, and Outlook

**Figure 13:**
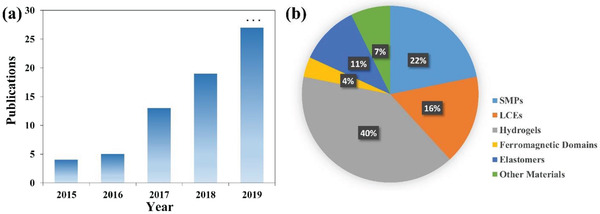
The summary statistics of publications about 4D printing by DIW. a) Numbers of publications versus year. b) Pie chart about categories of 4D printed materials by DIW.

We have seen that the DIW technique has been widely used in various materials, including SMPs, LCEs, and hydrogels, from pure materials to composites, single material to multimaterial, and micro to macro. **Figure** **13** summarizes the relevant data about the publications in recent five years. The publications about 4D printing by DIW have seen a significant increasing trend, as illustrated in Figure 13a. The main categories of 4D printed materials by DIW are depicted in Figure 13b, where shape‐shifting materials (i.e., hydrogels, SMPs, and LCEs) take up 78% in all publications. Hydrogels are the most used shape‐shifting materials at a percentage of 40%, followed by SMPs and LCEs, with the percentage of 22% and 16%, respectively. The open source of apparatus creates the conditions for printing different types of materials due to the convenience of setting temperatures of printing nozzles and platforms, small amount of materials, exchangeable barrels, and various nozzle sizes. The printing process requires moderate viscosity of printed ink and shear‐thinning performance during extrusion. If the ink is too dilute, it would not have shape retention capability during deposition. On the contrary, if it is too viscous, it would clog the syringe and the extruded filament would not be continuous.^[^ ^36^ ^]^ Even though, the regulation of viscosity is quite flexible for DIW because there have been many reported methods, such as dissolving in solvent,^[^ ^16^, ^56^, ^58^, ^60^ ^]^ printing at high or low temperature,^[^ ^61^, ^66^, ^80^, ^81^, ^82^, ^84^, ^119^ ^]^ adding rheology modifier,^[^ ^11^, ^61^, ^63^, ^64^, ^65^ ^]^ and UV‐assisted setup.^[^ ^16^, ^64^, ^80^, ^81^, ^82^, ^83^, ^84^, ^120^ ^]^ For thermoplastic SMPs, preparing a high viscous ink consisting of volatile solvents and dissolvable SMPs is essential to realize successful DIW printing. For example, PLA‐ and PLMC‐based SMPs and SMPCs were dissolved in dichloromethane, which was a good volatile solvent to endow the material with shear‐thinning performance.^[^ ^16^, ^56^, ^58^, ^60^ ^]^ Adding rheology modifier is another efficient method such as the viscosity of SMPU was adjusted by mixing with PEO or gelation.^[^ ^61^ ^]^ The gelatin was sensitive to temperature thus the printing temperature could be adjusted to obtain appropriate viscosity. As for thermoset SMPs, it usually requires addition of rheology modifier, postprocessing such as UV illumination during printing or thermal cure after printing, and regulation of printing temperature. For instance, the addition of CNF fillers,^[^ ^10^ ^]^ silica fillers and particles^[^ ^63^, ^64^, ^65^, ^68^ ^]^ endowed the ink with shear‐thinning behavior. Meanwhile, the introduction of photocurable acrylates under UV‐assisted printing followed by thermal cure also enhanced the shape retention ability of printed structures.^[^ ^64^, ^65^, ^68^ ^]^ The regulation of printing temperature enabled the ink to depolymerize to oligomers that facilitated extrusion from fine nozzles.^[^ ^66^ ^]^ When print LCEs, the printing temperature is set below *T*_NI_ (in nematic state) so that the ink exhibits shear‐thinning performance.^[^ ^80^, ^81^, ^82^, ^83^, ^84^, ^85^, ^86^ ^]^ After extrusion, UV light on the print head polymerizes the ink into crosslinking elastomers and locks the molecular alignment. The difference between printing and platform temperatures leads to a significant increase of viscosity and modulus of the material so that the printed structure could preserve the printed shape. Besides, rheology modifier enhances shear‐thinning performance of LCE ink, such as the addition of nanoclay enabled the ink to print at room temperature.^[^ ^85^ ^]^ As for hydrogels, both the physically or chemically crosslinking networks of hydrogels could be realized by introduction of rheology modifier,^[^ ^92^, ^93^, ^97^, ^98^, ^99^ ^]^ UV irradiation of photocurable monomers and postcuring.^[^ ^94^, ^95^, ^96^, ^97^, ^98^, ^99^, ^101^, ^102^, ^103^, ^104^, ^106^, ^108^, ^112^, ^113^, ^114^ ^]^ Besides, the viscosity varies in the sol–gel phase transition so that the printing temperature could also be adjusted to obtain appropriate viscosity.^[^ ^100^, ^102^, ^105^, ^106^, ^112^ ^]^ When printing elastomers, the viscosity of mixture comprising PDMS and curing agent is already suitable for DIW printing at room temperature directly.^[^ ^119^, ^123^, ^124^ ^]^ The addition of rheology modifier, such as fumed silica imparted the resin with a more significant shear‐thinning behavior.^[^ ^131^ ^]^ For ferromagnetic materials, the introduction of rheology modifier such as fumed silica achieved the required rheological properties.^[^ ^11^, ^120^ ^]^ Like the methods mentioned above, mixing with photocurable acrylates or elastomers with appropriate viscosity also meet the printing requirements.^[^ ^120^ ^]^ Besides, a magnetic field is necessary when printing ferromagnetic domains, which endows the printed filament with patterned magnetic polarity. The combination of prestretched elastomer with release of strain also creates the fourth dimension of time for commercial elastomers.

The application of 4D printed materials and structures based on DIW involves biomedical field, electronics, soft robotics, and smart actuators. For SMPs and SMPCs, the huge difference of modulus between glassy and rubbery state makes them excellent candidates for all these applications mentioned above. If SMPs consist of thermoplastic polymer with low *T*_g_ or possess reversible networks, they are inherently capable of self‐healing properties. When applied in biomedical field, SMPs or SMPCs need to be biodegradable and nontoxic to ensure safe requirement. Besides, adding some bioactive factors into printed ink endows printed structure with dynamic properties with the change of time. When applied in electronics, electrical response is often required thus the addition of conductive nanofillers or nanoparticles are necessary. The relatively large recovery stress produced in shape change of SMPs and SMPCs enables them to be applied in smart actuators and robotics. For LCEs, the two‐way shape transformation usually makes them candidate materials for smart grippers and soft actuators. The large reversible strain and mechanical actuation performance is the reason why LCEs are usually used in lifting loads, mimicking artificial muscles, and actuating self‐transformation. Besides, the different design of alignment in each layer created the conditions for complex shape transformation of whole structures. Hydrogels exhibit great potential in biomedical applications because hydrogel ink usually contains large content of water, which is beneficial for cellular activities. The internal structure within hydrogels makes them provide sufficient space for cell growth only by less materials. Besides, they are easily composited with cells thus providing bioactivity for tissue engineering. The significant volume change within the hydrogel structure upon multistimuli could also transfer energy which could be applied as soft grippers. The ferromagnetic domains could be accurately patterned and programmed under specific directions of magnetic field. The ultrafast and remotely actuated shape transformation makes them exhibit prospect in electronics, tissue engineering, and soft robotics. The elastomers with large stretchability accompanied by strain release always possess huge shape change, thus showing potential in soft robotics and actuators. The intrinsic ductility also endows them with excellent repeatability applied in electronics, when mixed with conductive nanofillers or nanoparticles.

However, there are still some challenges behind these great developments. First, although the DIW technique is friendly to various types of materials, the printing speed is relatively low and the interface is poor because of the layer‐by‐layer deposition. Thus the limitation of this printing technology needs to be broken in future to construct high‐resolution 4D structures with higher printing speed. Since the technique of continuous liquid interface production (CLIP) has broken the limits of DLP and realizes the fastest 3D printing speed,^[^ ^141^, ^142^ ^]^ no one knows whether the setup of DIW technique could be updated in future. Second, the applicable materials require more categories and more functionalities, which is the biggest challenge of 4D printing.^[^ ^41^ ^]^ The most commonly used materials for 4D printing by DIW are shape‐shifting materials due to their intrinsic shape‐changing capabilities. For SMPs and SMPCs, the shape transformation is not reversible, thus they usually require shape programming to be deformed into new shapes for next round of shape change. Especially when used as soft actuators, they could not perform repetitive shape change without external programming. However, some circumstance is not applicable for repeatable mechanical programming. Therefore, exploring two‐way SMPs and SMPCs is important for further extensive applications in 4D printing. It should be pointed out that some biomedical applications only require one‐time of shape change, thus the large recovery stress and fast shape transformation of SMPs may play an important role. For example, intravascular devices only require one‐way shape change with a large recovery stress and work capacity to support blood vessel or remove thrombus. Maybe this is also a direction that researchers could focus on regarding SMP‐based 4D structures in the future. For LCEs, they usually require high temperature to decrease the viscosity for smooth printing but the extruded state needs to be in nematic state for molecular alignment, which is contradictory. Besides, the *T*_NI_ of LCEs is relatively high, thus it usually requires high printing temperature and the temperature distribution may have some unknown effect on the order parameter of final printed object. Overall, the 4D printed LCEs show a relatively lower value of blocking stress (0.038–0.5 MPa) and work capacity (0.1–39 J kg^−1^) than nonprinted LCEs actuators (0.5–2.53 MPa).^[^ ^143^, ^144^, ^145^ ^]^ For example, the interpenetrating main‐chain LCE network fabricated by Lu et al. reached a maximum blocking stress and work capacity of 2.53 MPa and 1267.7 kJ m^−3^, respectively.^[^ ^145^ ^]^ Since the 4D printing of LCEs is just at an early stage, the materials selection of 4D printed LCE system is not as various as nonprinted LCEs. Thus exploring novel LCEs is an important trend to fulfill higher actuation performance toward application of robotics. Besides, printing single material of LCEs limit their applications in soft robotics and actuators as they only have function of lifting things. Thus exploring LCEs with low *T*_NI_ or printing them with other materials into multimaterial structure would doubtlessly broaden their functionality and actuation method toward soft robotics and biomedical area. Another challenge for LCEs is that the director patterns always lie in *x*–*y* plane, not in the vertical direction. Thus developing an omnidirectional director pattern is a trend of 4D printed LCEs. As for hydrogels, although the reversible shape transformation can be realized, the response time is consuming, especially for water‐responsive hydrogels whose shape change require swelling or deswelling in liquid. For example, the application of hydrogels in smart robotics and actuators is not as common as LCEs, though their actuation stress and work capacity were relatively better. In one hand, a relatively slow recovery rate takes too much time to fulfill behavior of grabbing or propelling objects. On the other hand, the shape transformation of water‐responsive hydrogels require liquid, which is not always applicable to stringent environment. This is the reason why 4D printed hydrogels are more frequently utilized in the field of tissue engineering because the above mentioned shortage could be avoided in this application. We envision that in the future, exploring other types of hydrogels is necessary for environment where liquid is not applicable. This would enable 4D printed hydrogel structures to play a more important role in soft robotics and actuators.

Based on these challenges and outlook of shape‐shifting materials, we propose that the range of materials applicable to DIW‐based 4D printing needs to grow. For example, new chemistries for crosslinking networks during or after printing would extend their applications in photochemical responses and harsh environment. Some materials are not stimuli‐responsive themselves and require additives to make them smart in corresponding stimuli. Although additives make them ease for external response, developing materials with inherent stimuli‐responsive properties is still important because some additives may interfere continuous printing as they are easy to agglomerate.^[^ ^146^ ^]^ As for multimaterial printing, most reported methods involve multiple nozzles, which is troublesome to avoid motion interference of ink supplies. Graded multimaterial may be achieved with one set of ink supply to program different material densities and preset stress at specific locations to simplify the manufacturing process in the future. Besides, it should be pointed out that the existing printing methods may not be applicable to new development of printed materials, thus the modification of printing setup according to new materials is also important to meet the demands. At last, establishing mathematical models for accurately prediction of complex shape change is highly demanding for 4D printing. It could also help optimize the printing parameters such as nozzle size, printing pathway and structural size of the printed structure. Based on the existing “static” shape, some researchers have reported some structural topology optimization models to obtain “dynamic” shape.^[^ ^24^, ^147^, ^148^, ^149^ ^]^ There is no doubt that these structural topology optimization would enrich and predict more complicated transformations of stimuli‐responsive materials. By designing printed lattices or directions, the complex out‐of‐plane deformation could be easily achieved just on the basis of in‐plane printing, which is hard if only depending on single materials. In future, we envision that printing multimaterial with multifunctionality combined with several printing methods and structural optimization would be the mainstream of 4D printing, which will definitely extend the practical applications.

**Table 1:** A summary of 4D printable materials via DIW, utilized setups, and the physical properties, actuated approaches, as well as shape‐changing mechanism of the obtained 4D structures

Materials	Type of materials	Printing conditions	Physical properties	Actuation method	Shape‐changing mechanism	Refs.
PLA/BP	SMP	UV‐assisted at RT	*T*_g_ ^a)^: 66 °C *R*_f_: >90% *R*_r_: ≈99%	Heat	Shape memory behavior	^[^ ^16^ ^]^
PLA/BP/Fe_3_O_4_	SMPC	UV‐assisted at RT	*T*_g_ ^a)^: 71 °C *R*_f_: >90% *R*_r_: ≈95%	Magnetic field	Shape memory behavior	^[^ ^16^ ^]^
PLA/Ag@CNF	SMPC	Print at RT	*T*_g_ ^a)^: ≈80 °C *R*_r_: >99% *σ* > 10^5^ S m^−1^	Electric field	Shape memory behavior	^[^ ^56^ ^]^
PLMC	SMP	Print at RT	*T*_g_ ^a)^: 49 °C *R*_f_: >99% *R*_r_: >99%	Heat	Shape memory behavior	^[^ ^58^ ^]^
PLMC/CNT	SMPC	Print at RT	*T*_g_ ^b)^: 69 °C *R*_f_: >99% *R*_r_: >96%	Electric field	Shape memory behavior	^[^ ^60^ ^]^
PLMC/ITX/EDB/CNT	SMPC	Print at RT and post‐UV cure	*T*_g_ ^b)^: 57 °C *E*: ≈1017 MPa *σ*_c_: ≈26 MPa *ε*_b_: ≈24%	Electric field	Shape memory behavior	^[^ ^60^ ^]^
SMPU/PEO/SPIO NPs	SMPC	Print at −30 °C	*T*_m_: 62 °C *R*_f_: 91–100% *R*_r_: 74–96%	Heat	Shape memory behavior	^[^ ^61^ ^]^
SMPU/gelatin/SPIO NPs	SMPC	Print at 5 °C	*T*_m_: 54 °C *R*_f_: 65–95% *R*_r_: 83–89%	Heat	Shape memory behavior	^[^ ^61^ ^]^
Polystyrene/chitosan/carbon black	SMPC	Print at RT on prestretched polystyrene	*T*_g_: 102 °C	NIR irradiation	Shape memory behavior	^[^ ^62^ ^]^
ESBO/BFDGE/CNFs	SMPC	Print at RT and postcure	*T*_g_ ^a)^: −30–110 °C *R*_f_: ≈98% *R*_r_: ≈95%	Heat, electric field	Shape memory behavior	^[^ ^10^ ^]^
PDMS/macroballon shell/SiO_2_	SMPC	Print at RT	*T*_g_ ^a)^: 44 and 113 °C *R*_r_: ≈100%	Heat	Shape memory behavior	^[^ ^63^ ^]^
Acrylates/epoxy/SiO_2_	SMPC	UV‐assisted print at RT and postcure	*T*_g_ ^b)^: ≈76 °C *R*_f_: >97% *R*_r_: ≈99%	Heat	Shape memory behavior	^[^ ^64^ ^]^
Urethane diacrylate/PCL/SiO_2_	SMPC	Print at RT with post‐UV irradiation layer‐by‐layer	*T*_m_: 55 °C *R*_f_: >93% *R*_r_: >83%	Heat	Shape memory behavior	^[^ ^65^ ^]^
DA‐reactive PU	Thermoreversible SMP	Print at 130 °C	*T*_m_: 45 °C *σ*_b_: ≈22 MPa *ε*_b_: ≈295%	Heat and NIR irradiation	Shape memory behavior and DA reaction	^[^ ^66^ ^]^
PEGDA/aliphatic urethane diacrylate/epon resin 862/2,4,6‐trisdimethylaminomethyl phenol/photoinitiator/SiO_2_	SMP	Photomask‐assisted print at RT	*T*_g_: 0–20 °C	Heat	Shape memory behavior	^[^ ^68^ ^]^
RM82/*n*‐butylamine/photointiator 369	LCE	UV‐assisted print at 85 °C and postcure	*T*_NI_: 105 °C *E*: 4–18 MPa	Heat	Nematic–isotropic transition	^[^ ^80^ ^]^
RM82/*n*‐butylamine/photointiator 651	LCE	UV‐assisted print at 50 °C and postcure	*T*_NI_: 95 °C *E*: 3 MPa	Heat	Nematic–isotropic transition	^[^ ^81^ ^]^
RM82/*n*‐butylamine/photointiator 369	LCE	UV‐assisted print at 70 °C and postcure	*T*_NI_: 110 °C	Heat	Nematic–isotropic transition	^[^ ^82^ ^]^
RM257/EDDT	LCE	UV‐assisted print at RT	*T*_NI_: 42 °C	Heat	Nematic–isotropic transition	^[^ ^83^ ^]^
RM82 and RM257/three thiol spacers/vinyl crosslinker/photointiator 369	LCE	UV‐assisted print at RT‐65 °C	*T*_NI_: ≈28–105 °C	Heat	Nematic–isotropic transition	^[^ ^84^ ^]^
RM257/EDDT/photoinitiator/nanoclay	LCE	Print at RT and poststretch under UV	*T*_NI_: 65 °C	Heat	Nematic–isotropic transition	^[^ ^85^ ^]^
2,2′‐(ethylenedioxy)diethanethiol/1,4‐bis‐[4‐(6‐acryloyloxy‐hexyloxy)benzoyloxy]‐2‐methylbenzene/1,4‐bis‐[4‐(3‐acryloyloxypropypropyloxy) benzoyloxy]‐2‐methylbenzene/1,3,5‐triallyl‐1,3,5‐triazine‐2,4,6(1*H*,3*H*,5*H*)‐trione/trimethylamine/butylated hydroxytoluene/Irgacure 651	LCE	UV‐assisted print at 26 °C and post‐UV irradiation	*T*_NI_: 24 °C	Heat	Nematic–isotropic transition	^[^ ^86^ ^]^
1,4‐bis‐[4‐(6‐acryloyloxyhexyloxy) benzoyloxy]‐2‐methylbenzene/*n*‐butyl amine/butylated hydroxytoluene/Irgacure 651	LCE	UV‐assisted print at 50–55 °C and post‐UV irradiation	*T*_NI_: 94 °C	Heat	Nematic–isotropic transition	^[^ ^86^ ^]^
Copolymer of 4,4′‐bis(6‐hydroxyhexyloxy)‐biphenyl, 4‐(6‐hydroxyhexyloxy)cinnamic acid and *p*‐coumaric acid	LCE	Print at 200 °C with platform at 10 °C and post‐UV irradiation	*T*_NI_: 66 °C	Heat	Nematic–isotropic transition and temperature difference along the thickness direction	^[^ ^87^ ^]^
Methacrylated alginate/methacrylated hyaluronic acid/mouse bone marrow stromal cells	Hydrogel	Print at RT and post‐crosslinking under green light	Compressive strain: 60%	Solvent	Ion exchange reaction	^[^ ^94^ ^]^
*α*‐CD/F127/2,2‐dimethoxy‐2‐phenylacetophenone/*N*‐vinylpyrrolidone	Hydrogel	Print at RT with post‐UV irradiation	*E*: 40 000 Pa	Solvent	Inter‐ring hydrogen‐bonding interactions	^[^ ^95^ ^]^
F127DA/sodium alginate/2,2‐dimethoxy‐2‐phenylacetophenone/methotrexate	Hydrogel	Print at RT with post‐UV irradiation	*R*_f_: ≈81% *R*_r_: ≈98%	Solvent	Alginate–Ca^2+^ coordination	^[^ ^96^ ^]^
Sodium carboxymethyl cellulose/pulp fibers/montmorillonite clay/citric acid	Hydrogel	Print at RT with post‐thermal crosslinking	*E*: 0.7–1.15 kPa	Solvent	Strain mismatch	^[^ ^97^ ^]^
Alginate/poly(*N*‐isopropylacrylamide) precursor	Hydrogel	UV‐assisted print at 10 °C	*T*_trans_: 32–35 °C	Heat	Ionic crosslinks	^[^ ^98^, ^99^ ^]^
Poly(*N*,*N*‐dimethylacrylamide)/NaCl/sodium dodecylsulfate	Hydrogel	Print at 45 °C	*T*_trans_: 30 °C	Heat	Reversible physical crosslinks	^[^ ^100^ ^]^
Poly(*N*‐isopropylacrylamide)/HEMA	Hydrogel	Print under UV irradiation	*T*_trans_: 32 °C	Heat	Thermally sol–gel transition	^[^ ^101^ ^]^
Agarose//laponite/acrylamide	Hydrogel	Print at 95 °C and post‐UV irradiation	*σ*_b_: 0.47 MPa *ε*_b_: 1258%	Heat	Thermally sol–gel transition of agarose	^[^ ^102^ ^]^
*N*‐isopropylacrylamide/acrylamide	Hydrogel	Print at 48 °C and post‐UV irradiation	–	Heat	Thermal‐responsive shrinking of *N*‐isopropylacrylamide	^[^ ^103^ ^]^
*N*‐isopropylacrylamide/PDMS/3‐(trimethoxysilyl)propyl methacrylate/MWCNT	Hydrogel	Print at RT and post‐UV irradiation	–	Light	Photothermal effect of MWCNTs	^[^ ^104^ ^]^
F127‐dimethacrylate/MWCNTs	Hydrogel	Print at RT in support gel	–	Light	Photothermal effect of MWCNTs	^[^ ^105^ ^]^
F127‐dimethacrylate/yeast cells	Hydrogel	Print at RT with post‐UV irradiation	*T*_trans_: 16.3 °C	Heat	Temperature dependent sol–gel phase transition	^[^ ^106^ ^]^
F127DA/PLGA/GO	Hydrogel	UV‐assisted print at RT	*R*_f_: >85 °C	NIR irradiation	Photothermal effect of GO	^[^ ^107^ ^]^
Alginate/PDA/cells	Hydrogel	Print with post‐UV irradiation	*E*: 0.19, 1.57 MPa	NIR irradiation	NIR‐induced hydration and dehydration	^[^ ^108^ ^]^
Polyacrylamide/poly(*N*‐isopropylacrylamide)/PEGDA/carbomer	Hydrogel	Print at RT with post‐UV irradiation	*T*_trans_: 35–37 °C	Heat, magnetic field	Sol–gel phase transition	^[^ ^112^ ^]^
Pluronic F127 diacrylate micelles/bacterial cells/chemicals	Hydrogel	Print at RT with post‐UV irradiation	Cell viability: >95%	–	Chemical diffusion over time	^[^ ^114^ ^]^
PDMS/ZrO_2_	Preceramic polymer (elastomer)	Print at 150 °C or RT and postcure	*σ*_c_: 547 MPa, s.g.: 1.6 g cm^−3^	Origami or prestrain	Restrictive deformation or release of elastic energy	^[^ ^119^ ^]^
PUA/HEMA/SiO_2_/alumina/photoinitiator	Ferromagnetic domains	UV‐assisted print with a magnetic field	*E*: ≈2–8 MPa, *σ*_c_: ≈4–8 MPa *ε*_b_: ≈150–380%, s.g.: 1.1 g cm^−3^	Ethyl acetate	Different swelling strains in multimaterial	^[^ ^120^ ^]^
NdFeB/PDMS/SiO_2_	Ferromagnetic domains	Print with a magnetic field	Power density: ≈22–309 kW m^−3^	Magnetic field	Magnetic polarity	^[^ ^11^ ^]^
Dragon Skin 10 SLOW	Elastomer	Print at RT	–	Prestrain	Release of elastic energy	^[^ ^123^ ^]^
PDMS/VO_2_	Elastomer	Print at RT	Phase transition temperature: 68 °C	Heat	Insulator–metal phase transition	^[^ ^124^ ^]^
rGO/CNT/LiCL/SMA substrate	SMA	Print at 95 °C and postcure	Phase transition temperature: 65 °C	Heat	Two‐way shape memory behavior	^[^ ^125^ ^]^
PLGA/CuSO_4_/bioactive liquid/gel	Biomaterial	Print at RT	*E*: 2.7–112.6 MPa, *σ*_c_: 0.8–9.5 MPa, *ε*_b_: 46.8–95.2%	Ethanol and water	Hydratation and salt leaching	^[^ ^126^ ^]^
Bisphenol A novolac epoxy/cyclopentanone	Commercial material	Print at RT	–	Liquid	Gradient distribution under UV	^[^ ^130^ ^]^
PDMS/glass fiber/SiO_2_	Elastomer	Print at RT	Δ*T* = −250 °C	Heat	Different coefficient of thermal expansion	^[^ ^131^ ^]^

a) *T*_g_: glass transition temperature tested by DSC; *R*_f_: shape fixity ratio; *R*_r_: shape recovery ratio; *σ*: electrical conductivity

b) *T*_g_: glass transition temperature tested by DMA; *E*: elastic modulus; *σ*_c_: tensile strength; *ε*_b_: strain at break; *T*_m_: melting temperature; *T*_NI_: nematic–isotropic temperature; *T*_trans_: transition temperature; RT: room temperature.

**Table 2:** The advantages and extensive applicability of DIW technique for printing various types of materials

Types of materials	Methods to make ink printable	Methods to retain shape	Potential applications	Representative refs.
Thermoplastic and partially crosslinking SMPs	Dissolve in volatile solvent Add rheology modifier Control printing temperature	High volatility of solvent	Biomedicine Electronics Smart grippers Soft actuator	^[^ ^16^, ^56^, ^58^, ^60^, ^61^ ^]^
Thermoset SMPs	Add rheology modifier Mix with photocurable acrylates Control printing temperature	UV illumination during printing Post‐thermal curing Photomask‐assisted	Electronics Biomedicine Soft actuators	^[^ ^10^, ^63^, ^64^, ^65^, ^66^, ^68^ ^]^
LCEs	Print below *T*_NI_ Add rheology modifier	UV illumination to lock alignment of mesogens	Smart grippers Soft actuators	^[^ ^80^, ^81^, ^82^, ^83^, ^84^, ^85^, ^86^, ^87^ ^]^
Hydrogels	Add rheology modifier Mix with UV curable monomers and postcuring Control printing temperature	UV illumination during printing Post‐thermal curing	Biomedicine Tissue engineering Soft grippers Soft actuators Skin‐like sensor	^[^ ^92^, ^93^, ^94^, ^95^, ^97^, ^98^, ^99^, ^100^, ^101^, ^102^, ^103^, ^104^, ^105^, ^106^, ^107^, ^108^, ^109^, ^112^, ^113^ ^]^
Elastomers	Print directly Add rheology modifier	Intrinsic properties of mixture	Soft robotics Soft actuators Electronics	^[^ ^119^, ^123^, ^124^, ^131^ ^]^
Ferromagnetic domains	Mix with photocurable acrylates Add rheology modifier Mix with printable elastomer	UV illumination during printing	Electronics Tissue engineering Soft robotics	^[^ ^11^, ^120^ ^]^

## 7 Conclusion

We summarize the achievements of 4D printing by direct ink writing technique in terms of various types of materials and their potential applications ranging from biomedical field to robotics. We point out the superiority of direct ink writing technique in constructing active structures and the relationship between printed materials, structural design with the mechanism of shape transformation. Different categories of materials with specific properties changing with time apply to corresponding applications, such as electronics, biomedical field, tissue engineering, and soft robotics. We also propose the future challenges and outlook based on these achievements. We believe in future, with the improvement of printing hardware, new types of materials applicable for 4D printing and accurate prediction of shape‐morphing structures based on theoretical analysis, it would broaden the fast development of 4D printing.

## Conflict of Interest

The authors declare no conflict of interest.
